# LD-transpeptidase-mediated cell envelope remodeling enables developmental transitions and survival in *Coxiella burnetii* and *Legionella pneumophila*

**DOI:** 10.1128/jb.00247-24

**Published:** 2025-01-23

**Authors:** Dipak Kathayat, Yujia Huang, Joee Denis, Benjamin Rudoy, Hana Schwarz, Jacob Szlechter

**Affiliations:** 1 Department of Population Medicine and Diagnostic Sciences, Cornell University 575043 , Ithaca, New York, USA; 2 Department of Microbiology, Cornell University 251789 , Ithaca, New York, USA; NCBI, NLM, National Institutes of Health, Bethesda, Maryland, USA

**Keywords:** LD-transpeptidases, *C. burnetii*, *L. pneumophila*, developmental transitions, PG, β-barrel tethering, host, survival, intrinsic resistance, RpoS, fatty acid transporter

## Abstract

*Coxiella burnetii* and *Legionella pneumophila* are two phylogenetically related bacterial pathogens that exhibit extreme intrinsic resistance when they enter into a dormancy-like state. This enables both pathogens to survive extended periods in growth-limited environments. Survival is dependent upon their ability to undergo developmental transitions into two phenotypically distinct variants, one specialized for intracellular replication and another for prolonged survival in the environment and host. We currently lack an understanding of the mechanisms that mediate these developmental transitions. Here, we performed peptidoglycan (PG) glycoproteome analysis and showed significant enrichment of PG structures catalyzed by LD-transpeptidases (LDTs) in the survival variants of *C. burnetii* and *L. pneumophila*. This is supported by the upregulation of LDTs, resulting in susceptibility to carbapenem antibiotics. Furthermore, deletion of the most upregulated LDT, *lpg*1386, in *L. pneumophila* significantly changes PG architecture, survival, and susceptibility to antibiotics. Significantly regulated by RpoS, a stationary-phase sigma factor, LDT-dependent PG remodeling is differentially activated by the host intracellular growth environment compared to axenic culture. In addition, β-barrel tethering, a newly discovered mechanism of LDT-mediated cell envelope stabilization, seems not to be specific to the survival variants. Interestingly, an outer membrane (OM) long-chain fatty acid transporter (Lpg1810) is tethered to PG in *L. pneumophila*. Collectively, these findings show that LDT-mediated PG remodeling is a major determinant of developmental transitions and survival in *C. burnetii* and *L. pneumophila*. Understanding this mechanism might inform new therapeutic approaches for treating chronic infections caused by these pathogens, as well as suggest new methods to decontaminate environmental reservoirs during outbreaks.

**IMPORTANCE:**

*Coxiella burnetii* and *L. pneumophila* cause Q Fever and Legionnaire’s disease in humans, respectively. There is a lack of effective treatments for fatal chronic infections caused by these pathogens, particularly chronic Q Fever. These bacteria survive long term in nutrient-limited environments by differentiating into phenotypically distinct survival variants. Our study revealed that LDTs, a group of PG remodeling enzymes, play a prominent role in the phenotypic differentiations of these bacteria. We show that LDT-targeting carbapenems are effective against the survival variants, thus demanding the exploration of carbapenems for treating chronic infections caused by these pathogens. We report the tethering of a unique OM fatty acid transporter to PG in *L. pneumophila* that could indicate a novel function of tethering, that is, the uptake of nutrient substrates. Homologs of this transporter are widely present in the Methylobacteriaceae family of bacteria known to survive in water systems like *Legionella*, thus suggesting a potentially conserved mechanism of bacterial survival in nutrient-limited environments.

## INTRODUCTION

Bacteria can survive environmental or host-imposed growth-limiting stresses by entering into a hardy, non-replicating state, often termed dormancy or quiescence (1, 2). One of the several strategies employed by bacteria to survive dormancy is cell envelope modification (1). The bacterial cell envelope is a complex multilayered structure that plays a multitude of adaptive and protective roles in response to always-changing surroundings in often hostile environments (3). The periplasmic space of the Gram-negative cell envelope contains peptidoglycan (PG) layer composed of repeating subunits of sugars (N-acetylglucosamine [GlcNAc]-N-acetylmuramic acid [MurNAc]) connected by the short peptides (L-alanine (A)-D-glutamic acid (E)-*meso*-diaminopimelic acid (*m*DAP)-D-alanine (A)-D-alanine (A)) (4). The remodeling of PG sugar and/or peptide moieties is considered a key to bacterial survival under diverse challenges in the environment and host (4). The cross-linking of short peptides in PG is catalyzed by either penicillin-binding proteins (PBPs) or LD-transpeptidases (LDTs) (5). PBPs, the target of β-lactam antibiotics, catalyze 4-3 cross-linking (5). LDTs primarily mediate 3-3 cross-linking but can perform very diverse functions (5). *E. coli* has six LDTs: (LdtA-F), LdtD and LdtE form 3-3 cross-links, with LdtA, LdtB, and LdtC stabilizing the cell envelope by tethering the outer membrane (OM)-anchored Braun’s lipoprotein (Lpp) to PG, and LdtF cleaves Lpp from PG (6–9). LDTs also catalyze exchange reactions to replace the terminal D-ala in tetrapeptides and incorporate non-canonical D-amino acids (NCAAs) (10). In addition, LDTs mediate the resistance to β-lactam antibiotics (11, 12), strengthen PG to survive the OM assembly defect (13), and also contribute to different aspects of bacterial life cycle and pathogenesis (14, 15). Importantly, LDTs are known to be inactivated by the carbapenem antibiotics in various bacterial pathogens (5).

A recent study discovered a new role of LDTs, where LDTs mediate the covalent attachment of β-barrel OM proteins to PG (aka β-barrel tethering) in various Lpp-deficient environmental proteobacteria such as *Coxiella burnetii*, *Legionella pneumophila*, and *Agrobacterium tumefaciens* (16). This study also uncovered that LDTs are enriched (≥10) in these environmental proteobacteria (16); however, the physiological relevance of LDT enrichment is not completely understood. In *C. burnetii*, LDT2 (Cbu0318) catalyzes the PG tethering of β-barrel OM proteins, BbpA and BbpB (16). A unique lipoprotein, LimB, is also tethered to PG in *C. burnetii* but the LDT mediating this tethering is not known yet (16). Furthermore, LDTs catalyze the PG tethering of β-barrel OM proteins, MOMPs, in *L. pneumophila* (16). The current consensus in the field is that β-barrel tethering provides stability to cell envelope (16–19), but further investigation is needed to fully understand its relevance in bacterial physiology.

*C. burnetii* and *L. pneumophila* are two phylogenetically related, Gram-negative, intracellular bacterial pathogens with significant public health importance (20–22). Both of these pathogens can survive in growth or nutrient-limited environments for extended periods of time because of their extreme intrinsic resistance when they enter into a dormancy-like state (20, 21). However, the mechanisms behind their survival in growth-limited environments are underexplored, and the appropriate models to study survival mechanisms that mimic their natural ecology are also lacking. It is well known that the survival of these pathogens in growth-limited environments depends on their ability to undergo developmental transitions into phenotypically distinct variants (20, 21). Among the phenotypically distinct variants, one is specialized for intracellular replication; the large-cell variant (LCV) in *C. burnetii* and the replicative form (RF) in *L. pneumophila* (20, 21). The other variant is specialized for prolonged survival in the environment and host; the small-cell variant (SCV) in *C. burnetii* and the transmissive form (TF) in *L. pneumophila* (20, 21). LCV and RF are metabolically active forms with a typical-looking Gram-negative cell envelope, whereas SCV and TF are dormant-like forms with unusually thick cell envelopes (16, 20, 23, 24). However, it is not completely understood how and what specific cell envelope remodeling occurs during the phenotypic differentiations of these pathogens to facilitate long-term environmental survival, thus allowing them to be a constant transmission threat to susceptible hosts.

Here, we performed a high-resolution glycoproteome analysis (25) to unravel the cell envelope architecture of replicating (LCV and RF) and survival (SCV and TF) variants of *C. burnetii* and *L. pneumophila*. LDTs are upregulated with the enrichment of LDT-catalyzed PG structures in the survival variants of both of these pathogens, resulting in susceptibility to carbapenem antibiotics. Significantly regulated by the RpoS sigma factor, LDT-dependent PG remodeling is differentially activated by the host intracellular growth environment compared to the axenic culture. Furthermore, the deletion of LDT, *lpg*1386, in *L. pneumophila* significantly changes its PG structure, survival, and susceptibility to antibiotics. Altogether, our study unveils the mechanism behind phenotypic differentiation in *C. burnetii* and *L. pneumophila* that could contribute to the development of new methods to decontaminate environmental reservoirs during outbreaks as well as rational therapeutic approaches to treat chronic infections caused by these pathogens.

## MATERIALS AND METHODS

### Bacterial strains and growth conditions

Wild-type *C. burnetii* Nine Mile Phase II (clone 4, RSA 439) and ∆*rpo*S mutant strains were used in this study (26, 27). Bacteria were grown microaerobically in ACCM-D broth at 37°C in 5% CO_2_ and 2.5% O_2_ (28) unless otherwise indicated. Wild-type *L. pneumophila* Lp02 and ∆*rpo*S:*:kan* mutant strains were used in this study (29). Bacteria were grown using CYET plates or AYET broth and incubated at 37°C until colonies developed or as indicated wherever relevant (29).

### Construction of ∆*lpg*1386 deletion mutant strain

*L. pneumophila* strain containing in-frame deletion (∆*lpg*1386) was generated using the allelic exchange method (30–32). The primers used to construct and verify deletion mutant strain are listed in Table S1. Genomic DNA extracted (illustra bacteria genomicPrep Mini Spin Kit, GE Healthcare) from Lp02 and ∆*lpg*1386 strains were sequenced for whole-genome analysis (Plasmidsaurus) to confirm the deletion (accession, Lp02: CP169562, ∆*lpg*1386: CP169561, BioProject: PRJNA1157187).

### Peptidoglycan extraction, purification, HPLC, and LC-MS/MS analysis

PG was extracted using the published protocol (16, 23). PG was extracted from the exponential-phase LCVs and stationary-phase SCVs of *C. burnetii*. LCVs and SCVs were generated by culturing *C. burnetii* in ACCM-D broth for 6 and 14 days, respectively, and validated using an anti-ScvA (an SCV-specific protein) antibody (28). PG was also extracted from the exponential phase RFs and post-exponential phase TFs of *L. pneumophila* (29). RFs were generated by culturing *L. pneumophila* to exponential-phase (OD_600_ ~0.7) in AYET broth with shaking (180 rpm) at 37°C. TFs were generated by pelleting and resuspending the exponential-phase culture in sterile tap water followed by incubation for 6 days at 37°C with shaking at 180 rpm. We confirmed that this culture model yields the transmissive phenotype using the flagellin subunit *flaA* (p*flaA-gfp*) reporter system (29, 33). RFs exhibited minimal GFP fluorescence, while TFs showed an approximately 10-fold increase in GFP fluorescence (data not shown). For comparison with wild type, PG was extracted from SCVs and TFs of ∆*rpo*S mutant strains of *C. burnetii* and *L. pneumophila*, respectively. PG was also extracted and compared between TFs of Lp02 and ∆*lpg*1386 strains of *L. pneumophila*. The analysis of solubilized muropeptides was performed by rp-HPLC (Agilent Technologies) using water-trifluoroacetic acid 0.1% (vol/vol) as buffer A and water-acetonitrile 25% (vol/vol)-trifluoroacetic acid 0.05% (vol/vol) as buffer B (23, 25, 34). A UHPLC (Vanquish Horizon) system coupled with a Q-Exactive HF Hybrid Quadrupole-Orbitrap mass spectrometer (Thermo Fisher Scientific) was used for the LC-MS/MS analysis (25).

### MS data deconvolution and PG data analysis

PMi-Byos-3.11 (Protein Metrics Inc.) version was used to deconvolute the MS data and extract the .ftrs file (25, 34). Xcalibur (Thermo Fisher Scientific) Qual Browser was used for viewing the chromatograms and spectra from .raw files. PMi-Byos-4.3 or PMi-Byos-4.5 versions were used to analyze the PG data (25, 34). We started analyzing the PG data through Byos by searching for PG monomers using a FASTA database containing recursive *E. coli* monomer peptide sequences (A, AE, AE*m*DAP, and AE*m*DAP containing 20 canonical amino acids in a recurring manner at position 4th and 5th). A fixed modification of +72.0848 was added to J (an unused letter that Byos sets to 100.0000 Da) to represent *m*DAP (mass  =  172.0848). Variable modifications of 480.1961 (GlcNAc-MurNAc) and 438.185 (GlcN-MurNAc) were enabled on the peptide N-terminus. Search tolerances of 10 parts per million (ppm) for precursor mass and 20 ppm for fragment mass tolerance were set. The manual score cut-off was set to 0 and a protein false discovery rate (FDR) of 1%. Searches were performed with multicore options: heavy and fully specific trypsin cleavage parameters. We then searched for the PG-tethered proteins using Byos by performing unbiased searches against bacterial proteomes (16). The variable modifications of 480.196 (GlcNAc-MurNAc)  + 372.1645 (AE*m*DAP) or 438.185 (GlcN-MurNAc)  + 372.1645 (AE*m*DAP) were set and permitted once per peptide on any residue within the peptide. Searches were performed using non-specific cleavage parameters and multicore options: heavy. Search tolerances (10 ppm for precursor mass and 20 ppm for fragment mass), manual score cut-off (0), and protein FDR (1%) were set as above.

We then used the PGFinder tool to analyze the PG data (25, 35, 36). The theoretical monoisotopic masses of PG monomers and N- and C-terminal periplasmic sequences of PG-tethered proteins identified through Byos were used to build the databases for PGFinder searches (16, 25, 35, 36). Individual theoretical masses contained in the databases were compared with observed masses in the experimental data sets. Any observed mass within 10 ppm tolerance was considered a match. The modifications accounted for include the presence of anhydro groups, deacetylated and O-acetylated sugars, amidated amino acids, and modifications resulted from N-acetylglucosaminidase and amidase activities (loss of GlcNAc and lack of peptide stems, respectively). In-source decay products (loss of GlcNAc) and Na^+^/K^+^ salt adducts were also included. The intensities of in-source decay products and salt adducts were combined with that from parent ions when found within close retention time (a 0.5 min time window).

### Ovine trophectoderm (oTr1) cell culture

To understand the PG remodeling in *C. burnetii* under conditions mimicking its natural ecology (21), we cultured *C. burnetii* in sheep conceptus-derived oTr1 cells and harvested the bacteria for PG analysis. oTr1 cells were cultured following the published protocol (37). When cells reached the near confluency, spent media was replaced with fresh incomplete oTr1 media (media containing no fetal bovine serum and penicillin-streptomycin solution) and incubated for 3 h. After 3 h, incomplete oTr1 media was removed and the cells were infected with *C. burnetii* (NMII, MOI ~ 10) suspended in fresh incomplete oTr1 medium. Cells were incubated with *C. burnetii* infection medium for 24 h at 37°C, 5% CO_2_ incubator. After 24 h, the *C. burnetii* infection medium was replaced with fresh incomplete oTr1 media supplemented with 5% fetal bovine serum and incubated for ~28 days to generate the SCVs of *C. burnetii*. SCV formation was validated using an anti-ScvA antibody. After 28 days, cells attached to the bottom were scraped with the scraper, and the whole flask content including spent media was filtered through a 1,000 mL, 0.1 µM filter unit (Thermo Fisher Scientific) to collect the SCVs. PG was then extracted from those collected SCVs as described above.

### Gene expression analysis

To quantify the expression of LDTs in *C. burnetii* and *L. pneumophila*, we performed gene expression analysis using RT-qPCR (38). Total RNA was extracted using Aurum Total RNA Fatty and Fibrous Tissue Kit (Bio-Rad) using the manufacturer’s instructions. Before RNA isolation, samples were mixed with RNAprotect Bacteria Reagent (Qiagen) for RNase inactivation and stabilization of RNA. RNA was isolated from LCVs and SCVs cultured in ACCM-D broth, SCVs harvested from oTr1 cells, RFs and TFs generated from the AYET-tap water culture model, and SCVs and TFs of ∆*rpo*S mutant strains of *C. burnetii* and *L. pneumophila*, respectively. RNA quantity and quality were measured using a NanoDrop Spectrophotometer. DNA traces were removed using a genomic DNA elimination mix (Qiagen) and a total of 2.5 µg of purified RNA was used to synthesize cDNA using RT^2^ First Strand Kit (Qiagen). RT-qPCR was performed using Maxima SYBR green/ROX qPCR master mix (Thermo Fisher Scientific) in a CFX96 Touch Real-Time PCR machine (Bio-Rad) or 7500 Fast Real-Time PCR system (Applied Biosystems), with a 60°C annealing temperature. The primers were designed using the PrimerQuest Tool (Table S1) and obtained from Integrated DNA Technologies. The data were normalized to the housekeeping genes, GAPDH, *gyr*A, *gyr*B, *rpo*A, *rpo*B, and *rec*A, and relative fold change was calculated using the *∆∆*CT method (39).

### SDS-PAGE and immunoblot analysis

To validate the presence or absence of PG-OM tethering, SDS-PAGE followed by immunoblot analysis was performed following the published protocol (16). For *C. burnetii*, bacterial pellets were harvested from SCVs grown in ACCM-D broth and oTr1 cells and resuspended in PBS containing 0.5% Triton X-100. Lysozyme (Sigma-Aldrich) was added at 100 µg/mL to the bacterial suspension and incubated at 37°C for 2 h. Lysozyme-treated and untreated samples were separated by SDS-PAGE using 12% Criterion XT Bis-Tris Precast gels (Bio-Rad) and transferred to a polyvinylidene fluoride (PVDF) membrane. Membranes were blocked using 5% skimmed milk powder in TBST buffer and incubated with anti-LimB and anti-BpbA antibodies (16). Immunoblots were washed with TBST, developed with Clarity Western ECL substrate (Bio-Rad), and visualized in a ChemiDoc MP Imaging System (Bio-Rad). For *L. pneumophila*, PG samples extracted (not subjected to enzymatic digestions) from RFs and TFs of Lp02 were used to detect MOMPs (Lpg2961 and Lpg1974) and Lpg1810 tethering using a published protocol (40). Anti-MOMPs (Lpg2961 and Lpg1974) and anti-Lpg1810 antibodies were generated at GenScript using keyhole limpet hemocyanin (KLH)-conjugated peptides as antigens (MOMPs epitope: CGWRHWHDVDHEWDW, Lpg1810 epitope: QSSMRHEFYGYSRLC). The epitopes were selected based on the OptimumAntigen Design Tool (GenScript) and AlphaFold structural predictions. Samples were separated by SDS-PAGE using 4%–20% Mini-PROTEAN TGX Tris-Glycine Precast gels (Bio-Rad) and transferred to PVDF membrane. Membranes were blocked as above and incubated with anti-MOMPs and anti-Lpg1810 antibodies. Immunoblots were washed, developed, and visualized as above. For the identification of proteins directly from SDS-PAGE gel, protein bands stained with SYPRO Ruby (Thermo Fisher Scientific) were excised from the gel and identified using standard mass spectrometry analysis (41).

### Beta-lactam susceptibility, SDS and EDTA sensitivity, and survival studies

To corroborate our hypothesis that LDT activation mediates the intrinsic resistance of survival variants, we compared the beta-lactams (amoxicillin, cephalexin, ceftriaxone, aztreonam, tebipenem, and meropenem) susceptibility and SDS and EDTA sensitivity among developmental variants of *L. pneumophila. L. pneumophila* was grown to RFs and TFs as described above, OD_600_ adjusted, incubated with twofold diluted concentrations of beta-lactams, SDS, and EDTA for 48 h, and then spot plated on a CYET agar plate. Susceptibility and sensitivity were also compared between TFs of Lp02 and ∆*lpg*1386 strains. For the survival comparison of Lp02 and ∆*lpg*1386 strains, bacteria grown in AYET broth were adjusted to the same OD_600_, resuspended in sterile tap water, and OD_600_ measurements were taken every 3 days until day 6.

### Computational and statistical analyses

The phylogenetic analysis of LDTs was performed using MEGA 11 software (42). The protein schematics were generated using the drawProteins R package (43). AlphaFold predictions were run using Google Colab AlphaFold2 (44). The mutation analysis of the bacterial whole genomes was conducted using *breseq* 0.38.1 (SeqCenter, LLC) (45). The statistical analysis of the data sets generated in this study was performed using GraphPad Prism 9.5.0. The densitometry analysis of the immunoblot images was performed using ImageJ. All experiments were conducted in at least three biological replicates.

## RESULTS

### LDT-catalyzed PG structures are abundant in the survival variants of *C*. *burnetii* and *L*. *pneumophila*

**Fig 1:**
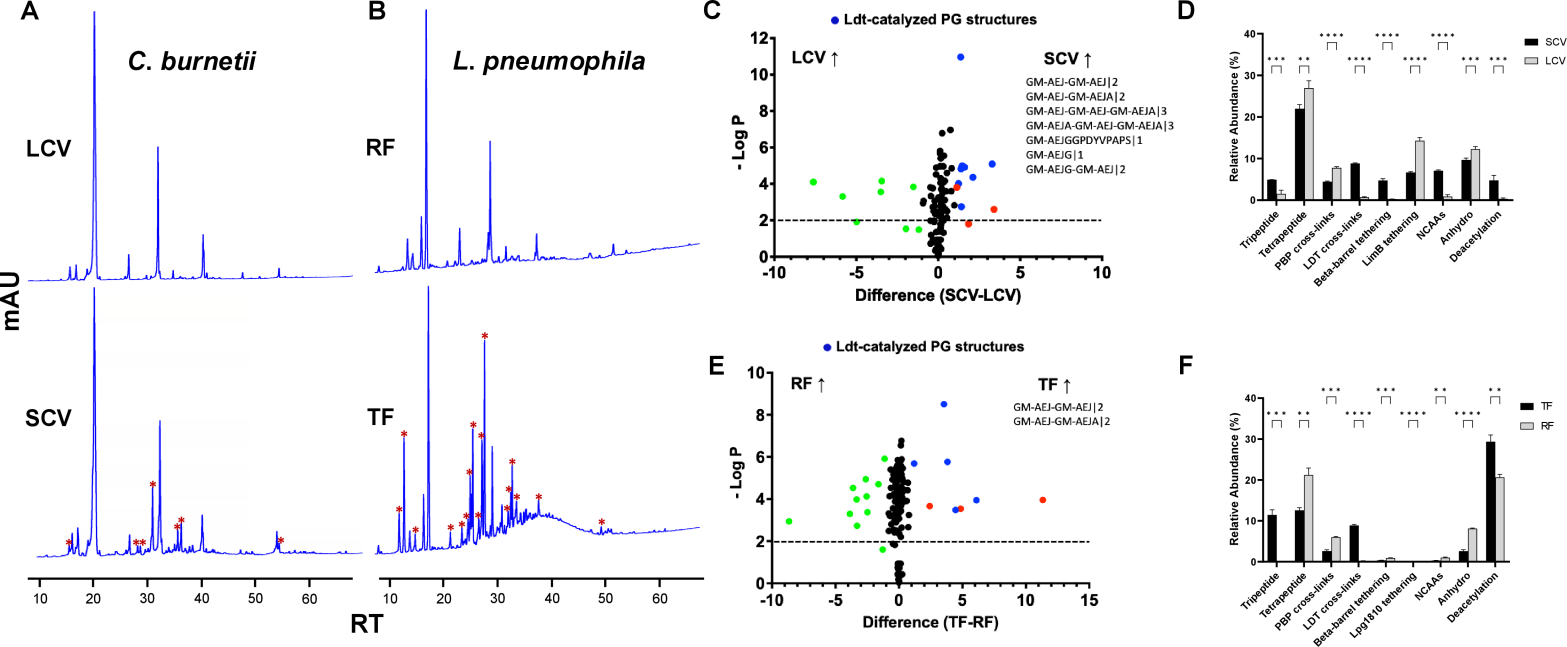
LDT-catalyzed PG structures are enriched in the survival variants of *C. burnetii* and *L. pneumophila*. (A and B) representative HPLC chromatograms of the replicating (LCV and RF) and survival (SCV and TF) variants of *C. burnetii* (**A**) and *L. pneumophila* (**B**), respectively. Asterisk “*” symbols indicate peaks unique to the survival variants. (C and E) volcano plots showing PG structures significantly abundant in the survival variants of *C. burnetii* (**C**) and *L. pneumophila* (**E**), respectively, compared to their replicating variants. PG structures with abundance >0.10% were only considered for this analysis. Blue dots indicate significantly abundant (*P <* 0.01 and >1% change in relative abundance) PG structures in the survival variants that are LDT-catalyzed, red dots indicate significantly abundant PG structures in the survival variants that are not LDT-catalyzed, and green dots indicate PG structures significantly abundant in the replicating variants. The PG structures denoted in the figures are LDT-catalyzed canonical PG structures which are significantly abundant in the survival variants. (D and F) relative abundance of PG structures in the replicating and survival variants of *C. burnetii* (**D**) and *L. pneumophila* (**F**), respectively. **P < 0.05, ***P < 0.01, ****P < 0.001, multiple unpaired t-tests.

We extracted PG from the two developmental variants of *C. burnetii* (LCVs and SCVs) and *L. pneumophila* (RFs and TFs) and performed mass spectrometry analysis to compare the PG architecture. The HPLC analysis of PG showed distinct new peaks in SCVs and TFs (survival variants) compared to LCVs and RFs (replicating variants), suggesting the remodeling of PG in the survival variants of *C. burnetii* and *L. pneumophila* (Fig. 1A and B). The annotation of PG structures by LC-MS/MS analysis (Fig. S1 and S2) revealed that LDT-catalyzed structures are abundant in the survival variants of both *C. burnetii* and *L. pneumophila* compared to their replicating variants (Fig. 1C and E). The LDT-catalyzed PG structures that were abundant in the survival variants of *C. burnetii* are those that mediate 3-3 cross-linking (GM-AEJ-GM-AEJ|2, GM-AEJ-GM-AEJA|2, GM-AEJ-GM-AEJ-GM-AEJA|3, and GM-AEJA-GM-AEJ-GM-AEJA|3), BbpA tethering (GM-AEJGGPDYVPAPS|1), and incorporation of NCAAs (GM-AEJG|1 and GM-AEJG-GM-AEJ|2) (Fig. 1C; Table S2). Similarly, the LDT-catalyzed PG structures that were abundant in the survival variants of *L. pneumophila* are those that mediate 3-3 cross-linking (GM-AEJ-GM-AEJ|2 and GM-AEJ-GM-AEJA|2) (Fig. 1E; Table S3). No significantly abundant PG structures catalyzed by LDTs were detected in the replicative forms (LCVs/RFs) of these pathogens. The PBPs-catalyzed PG structures that mediate 4-3 cross-linking (GM-AEJA-GM-AEJA|2) were abundant in the replicating variants of both *C. burnetii* and *L. pneumophila* (Fig. 1D and F; Tables S2 and S3). In addition, PG structures corresponding to β-barrel tethering, NCAAs incorporation, tripeptide (GM-AEJ|1), and deacetylation of PG sugars were significantly more abundant in the survival variants of *C. burnetii*, whereas significantly lesser LimB tethering (GM-AEJAKL|1), tetrapeptide (GM-AEJA|1), and anhydro PG sugars were found compared to its replicating variants (Fig. 1D; Table S2). Similar to *C. burnetii*, PG structures corresponding to tripeptide and deacetylation of PG sugars were significantly more abundant in the survival variants of *L. pneumophila*, whereas significantly lesser tetrapeptide and anhydro PG sugars were found compared to its replicating variants (Fig. 1F; Table S3). In contrast to *C. burnetii*, PG structures corresponding to β-barrel tethering and NCAAs incorporation were significantly more abundant in the replicating variants of *L. pneumophila* compared to its survival variants (Fig. 1F; Fig. S3; Table S3). Interestingly, we found an OM long-chain fatty acid transporter (Lpg1810) tethered to PG (GM-AEJKTPAPPEKDIT) in the survival variants of *L. pneumophila* (Fig. S3; Table S3, described later in detail).

### LDTs are upregulated in the survival variants of *C*. *burnetii* and *L*. *pneumophila*, resulting in susceptibility to tebipenem, a carbapenem antibiotic

**Fig 2:**
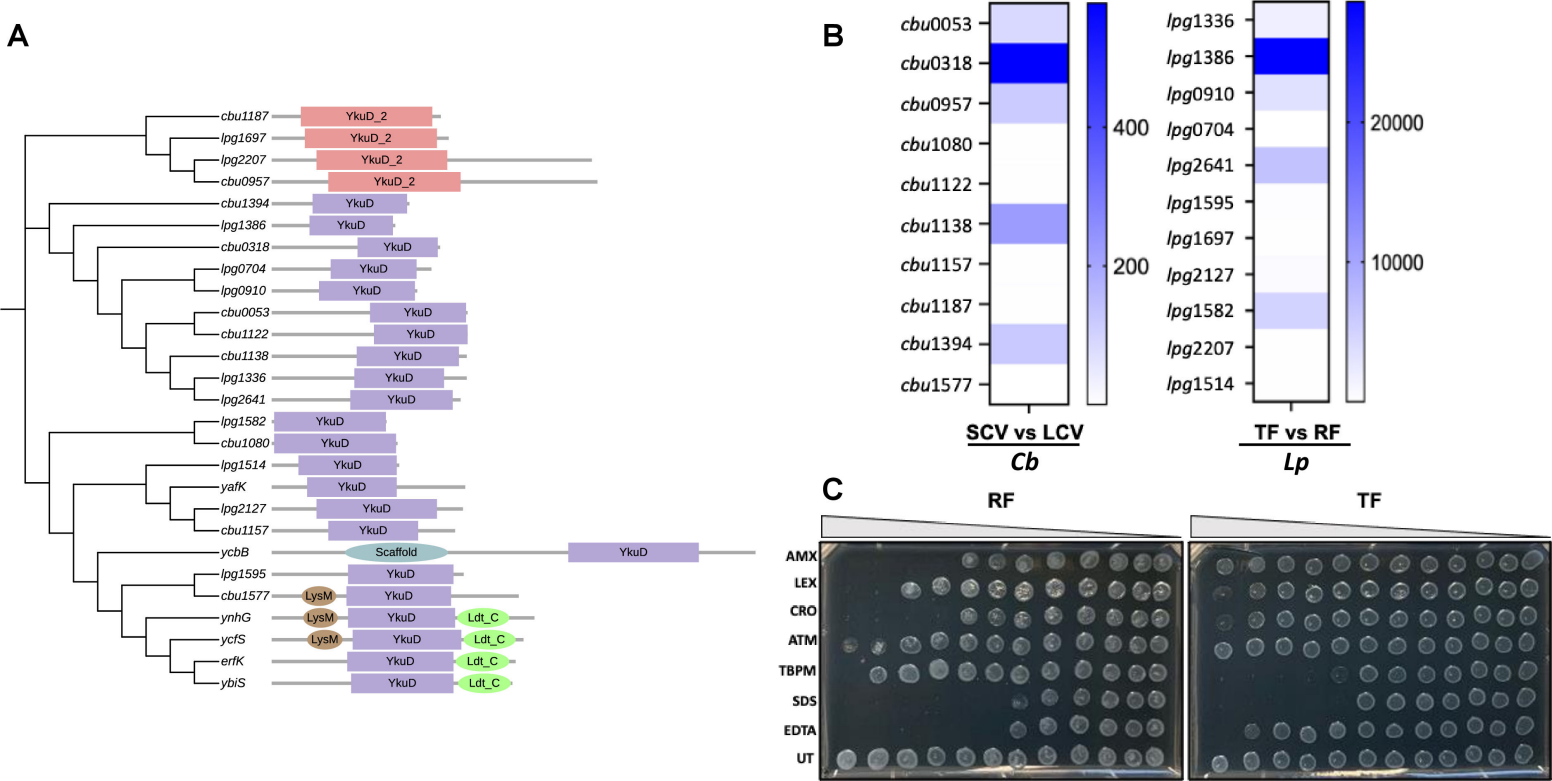
LDTs are upregulated in the survival variants of *C. burnetii* and *L. pneumophila* resulting in susceptibility to tebipenem, a carbapenem antibiotic. (**A**) Phylogenetic and protein domain analysis of LDTs of *E. coli*, *C. burnetii*, and *L. pneumophila.* DNA and protein sequences of *E. coli* (strain K12), *C. burnetii* (strain RSA493/Nine Mile Phase I), and *L. pneumophila* subsp. *pneumophila* (strain Philadelphia 1/ATCC 33152/DSM 7513) were used for the analysis. Colors in the protein schematics refer to the InterPro annotated protein domains. (**B**) Fold increase in expression of LDTs in the survival (SCV and TF) variants as compared to the replicating (LCV and RF) variants of *C. burnetii* (Cb) and *L. pneumophila* (Lp), respectively, as determined by RT-qPCR. The expression level of LDTs in the replicating variants is considered a baseline expression to calculate the fold increase in expression in the survival variants. (**C**) Susceptibility of the replicating and survival variants of *L. pneumophila* to β-lactam (amx: amoxicillin, lex: cephalexin, cro: ceftriaxone, atm: aztreonam) and carbapenem (tbpm: tebipenem) antibiotics, SDS, and EDTA. UT: untreated.

*C. burnetii* and *L. pneumophila* genomes contain 10 and 11 genes encoding diverse LDTs, respectively (Fig. 2A). Most of these LDTs, except *cbu*0957, *cbu*1080, and *lpg*1582, have signal peptides as predicted by SignalP 6.0 (46). To support the PG results, we measured and compared the expression of all predicted LDTs in two developmental variants of *C. burnetii* (LCVs and SCVs) and *L. pneumophila* (RFs and TFs). We found five LDTs upregulated in SCVs (*cbu*0318, *cbu*1138, *cbu*1394, *cbu*0957, and *cbu*0053) (Fig. 2B; Table S4) and highly upregulated in TFs (*lpg*1386, *lpg*2641, *lpg*1582, *lpg*0910, and *lpg*1336) (Fig. 2B; Table S4), providing the evidence of LDTs activation in the survival variants of these bacteria. The culture model used to generate TFs (sterile tap water) is likely more extreme in terms of nutritional limitations, as well as the number and types of stressors, compared to the ACCM-D broth used for generating SCVs. This is likely the primary reason for the drastic changes in the expression of LDTs observed in *L. pneumophila* compared to *C. burnetii*. We next tested the susceptibility of *L. pneumophila* developmental variants to LDT-targeting carbapenem and PBP-targeting β-lactam antibiotics. RFs were susceptible to β-lactam antibiotics (amoxicillin, cephalexin, and ceftriaxone), whereas TFs were resistant to those antibiotics (Fig. 2C; Table S5). Interestingly, tebipenem was four times more effective against TFs (MIC: 250 µg/mL) than to RFs (MIC: 1000 µg/mL) (Fig. 2C; Table S5). Furthermore, meropenem was also found effective against TFs (MIC: 50 µg/mL) (see Fig. 5B, Table S5), suggesting the enrichment of LDTs in the survival variants of *L. pneumophila*. In addition, TFs were less susceptible to SDS and EDTA than RFs (Fig. 2C; Table S5), providing further evidence of cell envelope remodeling in the survival variants of *L. pneumophila*.

### RpoS significantly contributes to the regulation of LDT-dependent PG remodeling in the survival variants of *C*. *burnetii* and *L*. *pneumophila*

**Fig 3:**
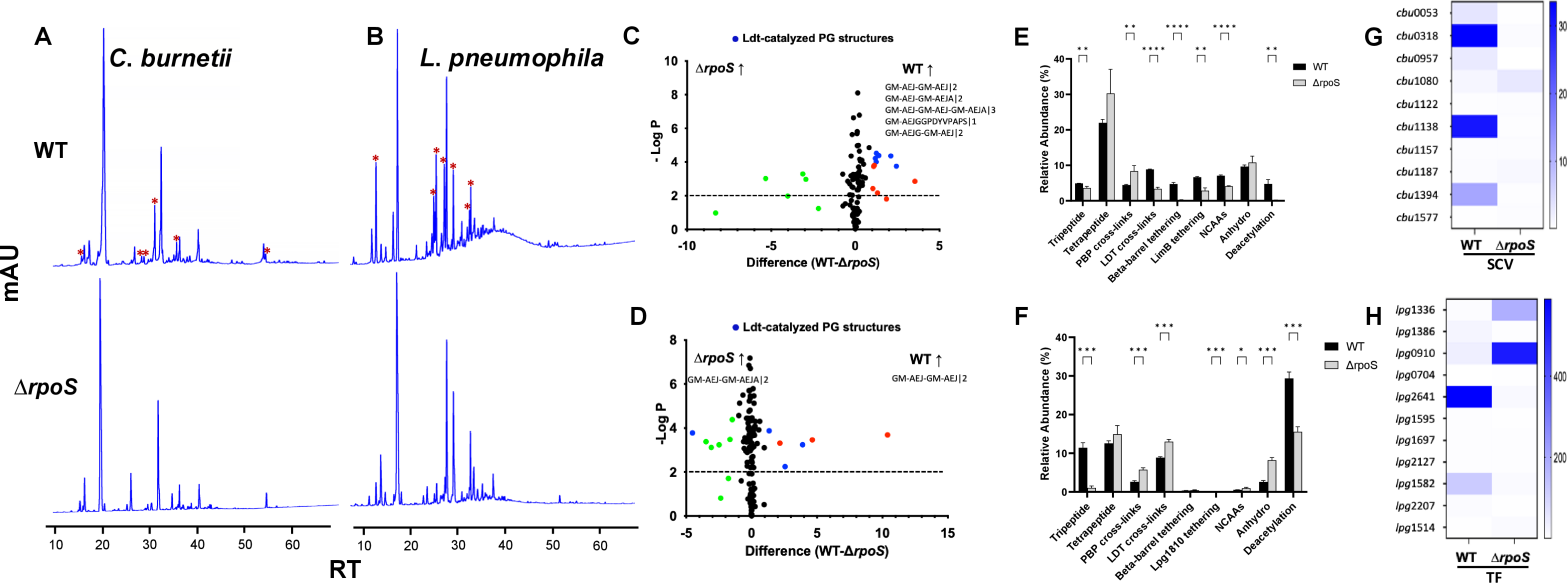
RpoS significantly contributes to the regulation of LDT-catalyzed PG remodeling in the survival variants of *C. burnetii* and *L. pneumophila*. (A and B) Representative HPLC chromatograms of the survival (SCV and TF) variants of wild-type (WT) and *rpoS* knock-out (∆*rpoS*) strains of *C. burnetii* (**A**) and *L. pneumophila* (**B**). Asterisk “*” symbols indicate peaks present in WT but absent in the ∆*rpoS* strain. (C and D) Volcano plots showing PG structures are significantly abundant in WT strains of *C. burnetii* (**C**) and *L. pneumophila* (**D**) compared to their ∆*rpoS* strains. PG structures with abundance >0.10% were only considered for this analysis. Blue dots indicate PG structures significantly abundant (*P <* 0.01 and >1% change in relative abundance) in WT/∆*rpo*S strain that are LDT-catalyzed, red dots indicate PG structures significantly abundant in WT strain that are not LDT-catalyzed, and green dots indicate PG structures significantly abundant in ∆*rpoS* strain. The PG structures denoted in the figures are canonical PG structures that are LDT-catalyzed and significantly abundant in WT/∆*rpo*S strains. (E and F) Relative abundance of PG structures in the survival variants of WT and ∆*rpoS* strains of *C. burnetii* (**E**) and *L. pneumophila* (**F**), respectively. *P < 0.1, **P < 0.05, ***P < 0.01, ****P < 0.001, multiple unpaired t-tests. (G and H) Fold change in expression of LDTs in the survival variants of WT and ∆*rpoS* strains of *C. burnetii* (**G**) and *L. pneumophila* (**H**), respectively, as determined by RT-qPCR.

We were interested in determining how LDT-catalyzed PG remodeling is regulated in *C. burnetii* and *L. pneumophila* and if it is regulated by RpoS, a stationary-phase/stress sigma factor (47). RpoS regulon in *E. coli* and other Gram-negative bacteria plays a central role in survival and adaptation under diverse environmental challenges through the activation of numerous regulatory processes (47). Furthermore, RpoS is known to have a role in developmental transitions and survival under nutrient-limited environments in *C. burnetii* and *L. pneumophila* (27, 48–50). We analyzed the PG extracted from the survival variants (SCVs and TFs) of ∆*rpo*S strains of *C. burnetii* and *L. pneumophila*. The HPLC (Fig. 3A) and mass spectrometry analysis revealed that LDT-catalyzed PG structures that mediates the 3-3 cross-linking (GM-AEJ-GM-AEJ|2, GM-AEJ-GM-AEJA|2, and GM-AEJ-GM-AEJ-GM-AEJA|3), BbpA tethering (GM-AEJGGPDYVPAPS|1), and incorporation of NCAAs (GM-AEJG-GM-AEJ|2) were less abundant in a ∆*rpo*S mutant of *C. burnetii* compared to its isogenic wild type (Fig. 3C; Fig. S4; Table S6). In *L. pneumophila*, LDT-catalyzed PG structure that mediates the 3-3 cross-linking (GM-AEJ-GM-AEJ|2) was less abundant in a ∆*rpo*S mutant compared to its isogenic wild type (Fig. 3B and D; Fig. S5; Table S7). The PBPs-catalyzed PG structure that mediate 4-3 cross-linking (GM-AEJA-GM-AEJA|2) was more abundant in the ∆*rpo*S mutants of both *C. burnetii* and *L. pneumophila* (Fig. 3E and F; Tables S6 and S7). Importantly, while no LDT-catalyzed PG structures were present in a ∆*rpo*S mutant of *C. burnetii*, one LDT-catalyzed PG structure (GM-AEJ-GM-AEJA|2) was still abundantly present in a ∆*rpo*S mutant of *L. pneumophila* (Fig. 3D; Fig. S5; Table S7), suggesting that RpoS only partially regulates the LDT-dependent PG remodeling in the survival variants of *L. pneumophila* but significantly regulates them in *C. burnetii*. In addition, PG structures corresponding to β-barrel tethering, LimB tethering, NCAAs incorporation, tripeptide, and deacetylation of PG sugars were significantly more abundant in the wild-type *C. burnetii* compared to its ∆*rpo*S mutant (Fig. 3E). Similar to *C. burnetii*, tripeptide and deacetylated PG sugars were significantly more abundant in the wild-type *L. pneumophila* compared to its ∆*rpo*S mutant (Fig. 3F). Furthermore, PG structures corresponding to Lpg1810 tethering were significantly more abundant in the wild-type *L. pneumophila*, whereas significantly lesser anhydro PG sugars and incorporation of NCAAs were found compared to its ∆*rpo*S mutant (Fig. 3F; Table S7). Supporting the PG results above, none of the LDTs upregulated in the survival variants of the wild type were upregulated in a ∆*rpo*S mutant of *C. burnetii* (Fig. 3G; Table S4). Whereas, among the five LDTs upregulated in the survival variants of *L. pneumophila* (*lpg*1386, *lpg*2641, *lpg*1582, *lpg*0910, and *lpg*1336), three LDTs (*lpg*1386, *lpg*2641, and *lpg*1582) were downregulated and two LDTs (*lpg*0910 and *lpg*1336) were upregulated in a ∆*rpo*S mutant of *L. pneumophila* (Fig. 3H; Table S4). These findings thus demonstrate that RpoS significantly contributes to the regulation of LDT-dependent PG remodeling in the survival variants of *C. burnetii* and *L. pneumophila*.

### Host intracellular growth environment elevates the LDT-catalyzed PG remodeling and alters the tethering systems in *C*. *burnetii*

**Fig 4:**
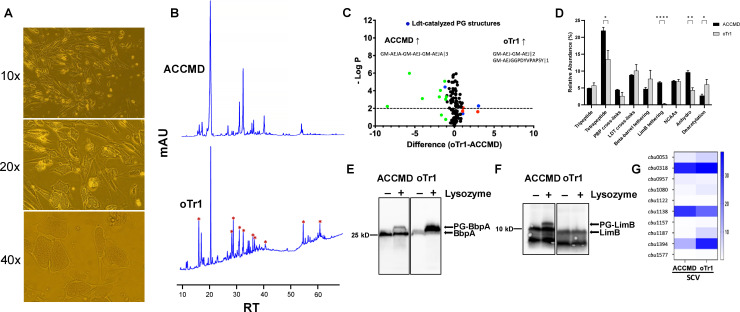
Host intracellular growth environment differentially activates the LDTs, alters tethering systems, and elevates LDT-catalyzed PG remodeling in *C. burnetii*. (**A**) Microscopy images showing heterogenous *Coxiella* containing vacuoles (CCVs) in infected oTr1 cells. Images were taken using Olympus CKX53 inverted microscope at 10×, 20×, and 40× magnifications at day 14 post-infection. (**B**) Representative HPLC chromatograms of the survival (SCV) variants of *C. burnetii* harvested from ACCM-D broth and oTr1 cells. Asterisk “*” symbols indicate peaks whose abundance is drastically changed in SCVs from oTr1 cells compared to ACCM-D broth. (**C**) Volcano plot showing PG structures significantly abundant in SCVs from oTr1 cells compared to ACCM-D broth. PG structures with abundance >0.10% were only considered for this analysis. Blue dots indicate significantly abundant (*P <* 0.01 and >1% change in relative abundance) PG structures in SCVs from oTr1 cells/ACCM-D broth that are LDT-catalyzed, red dots indicate significantly abundant PG structures in SCVs from oTr1 cells that are not LDT-catalyzed, and green dots indicate PG structures significantly abundant in SCVs from ACCM-D broth. The PG structures denoted in the figure are LDT-catalyzed canonical PG structures which are significantly abundant in SCVs from oTr1 cells/ACCM-D broth. (**D**) Relative abundance of PG structures in SCVs from oTr1 cells compared to ACCM-D broth. *P < 0.1, **P < 0.05, ****P < 0.001, multiple unpaired t-tests. (**E**) Immunoblot images showing relative quantitation of BbpA tethering in SCVs from oTr1 cells and ACCM-D broth. Whole-cell lysates were either untreated or treated with lysozyme and probed with anti-BbpA antibody. Densitometry analysis calculates PG tethered BbpA 2.4 times higher in SCVs from oTr1 cells compared to ACCM-D broth. (**F**) Immunoblot images showing relative quantitation of LimB tethering in SCVs from oTr1 cells and ACCM-D broth. Whole-cell lysates were either untreated or treated with lysozyme and probed with anti-LimB antibody. (**G**) Fold change in expression of LDTs in SCVs from oTr1 cells and ACCM-D broth determined by RT-qPCR.

We were interested in investigating whether the intracellular growth of *C. burnetii* also leads to LDT-mediated PG remodeling. To mimic the natural ecology of *C. burnetii* (21), we used oTr1 cells derived from the sheep conceptus for intracellular growth of *C. burnetii* (37) and analyzed the PG of SCVs harvested from these cells. *C. burnetii* makes massive vacuoles of heterogenous sizes inside oTr1 cells (Fig. 4A). The HPLC analysis showed drastically different peaks in SCVs harvested from oTr1 cells as compared to SCVs from ACCM-D broth (Fig. 4B; Fig. S6). The PG analysis showed that SCVs from oTr1 cells had increased LDT-catalyzed 3-3 cross-linking (GM-AEJ-GM-AEJ|2) and BbpA tethering (GM-AEJGGPDYVPAPSY|1), but decreased PBPs-catalyzed 4-3 cross-linking (GM-AEJA-GM-AEJA|2) and completely absent LimB tethering (Fig. 4C and D; Table S8). The tethering differences between the oTr1- and ACCM-D-derived SCVs were subsequently validated by the immunoblot analyses, where significantly increased PG tethered BbpA (Fig. 4E) and no PG tethered LimB (Fig. 4F) was detected in oTr1-derived SCVs compared to SCVs from ACCM-D broth. In addition, oTr1-derived SCVs had an increased abundance of tripeptide and deacetylated PG sugars, while decreased tetrapeptide and anhydro PG sugars were found (Fig. 4D). Interestingly, we found seven LDTs upregulated in oTr1-derived SCVs, also in different magnitudes than ACCM-D-derived SCVs (oTr1: *cbu*0318 > *cbu*1394 > *cbu*1138 > *cbu*0053 > *cbu*1187 > *cbu*1080 > *cbu*0957; ACCM-D: *cbu*0318 > *cbu*1138 > *cbu*1394 > *cbu*0957 > *cbu*0053) (Fig. 4G; Table S4), suggesting a substantial role of growth environment in the LDTs activation in bacteria.

### Deletion of the most upregulated LDT, *lpg*1386, in *L*. *pneumophila* resulted in significant changes in PG structure, survival, and susceptibility to antibiotics

**Fig 5:**
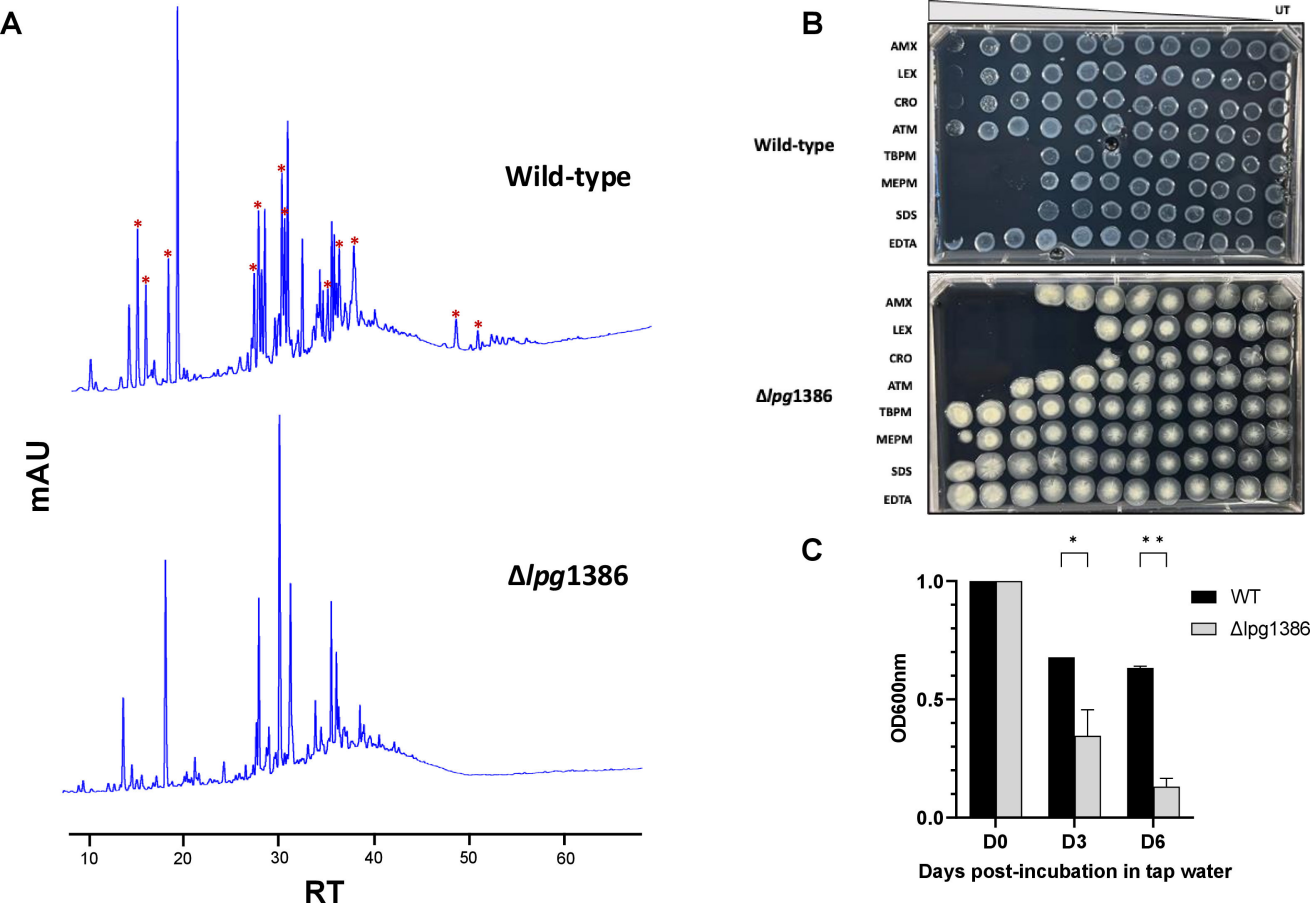
Deletion of the most upregulated LDT, *lpg*1386, in *L. pneumophila* significantly changes the PG structure, survival, and susceptibility to antibiotics. (**A**) Representative HPLC chromatograms of the survival (TF) variants of wild-type (WT) and *lpg*1386 knock-out (∆*lpg*1386) strains of *L. pneumophila*. Asterisk “*” symbols indicate peaks present in WT but absent in the ∆*lpg*1386 strain. (**B**) Susceptibility of TFs of WT and ∆*lpg*1386 strains of *L. pneumophila* to β-lactam (amx: amoxicillin, lex: cephalexin, cro: ceftriaxone, atm: aztreonam) and carbapenem (tbpm: tebipenem, mepm: meropenem) antibiotics, SDS, and EDTA. UT: untreated. (**C**) Survival of WT and ∆*lpg*1386 strains in tap water was monitored by measuring OD_600_ nm. *P < 0.1, **P < 0.05, multiple unpaired t-tests.

Knowing that multiple LDTs are upregulated in *C. burnetii* and *L. pneumophila* upon differentiation into the survival variants, we aimed to understand the specific functions of LDTs by making genetic knockouts. We deleted the most significantly upregulated LDT, *lpg*1386, from the *L. pneumophila* genome. The mutation analysis of the whole-genome sequences confirmed no other mutations other than the 340 bp deletion of *lpg*1386. Upon HPLC and mass spectrometry analysis of the PG extracted from TFs of ∆*lpg*1386 mutant, we found that the deletion of *lpg*1386 resulted in significant changes in PG structure, mainly loss of PG structures corresponding to LDT-catalyzed 3-3 cross-linking (GM-AEJ-GM-AEJ|2, GM-AEJ-GM-AEJ-GM-AEJ|3, and GM-AEJ-GM-AEJ-GM-AEJA|3) (Fig. 5A; Fig. S7), demonstrating the role of *lpg*1386 in 3-3 cross-linking in *L. pneumophila*. In addition, TFs from a ∆*lpg*1386 mutant were more susceptible to β-lactam antibiotics (amoxicillin, cephalexin, ceftriaxone, and aztreonam) compared to its wild type, but resistant to carbapenem antibiotics (tebipenem and meropenem) unlike wild type (Fig. 5B; Table S5). Surprisingly, the ∆*lpg*1386 mutant showed higher resistance to SDS than the wild type (Fig. 5B; Table S5) which might be due to changes in other components of the bacterial cell envelope, such as lipids, caused by the deletion of *lpg*1386. Furthermore, ∆*lpg*1386 survived significantly less well in tap water compared to its wild type (Fig. 5C; Fig. S8). The deletion of *lpg*1386 however resulted in a drastic change in colony morphology which requires further investigation (Fig. 5B; Fig. S8). These results suggest that LDT, *lpg*1386, has a significant role in maintaining cell envelope integrity, protection against environmental threats and/or stressors, and survival under nutrient-limited environments.

### β-barrel tethering is not limited to the survival variants and OM long-chain fatty acid transporter is tethered to PG in *L*. *pneumophila*

**Fig 6:**
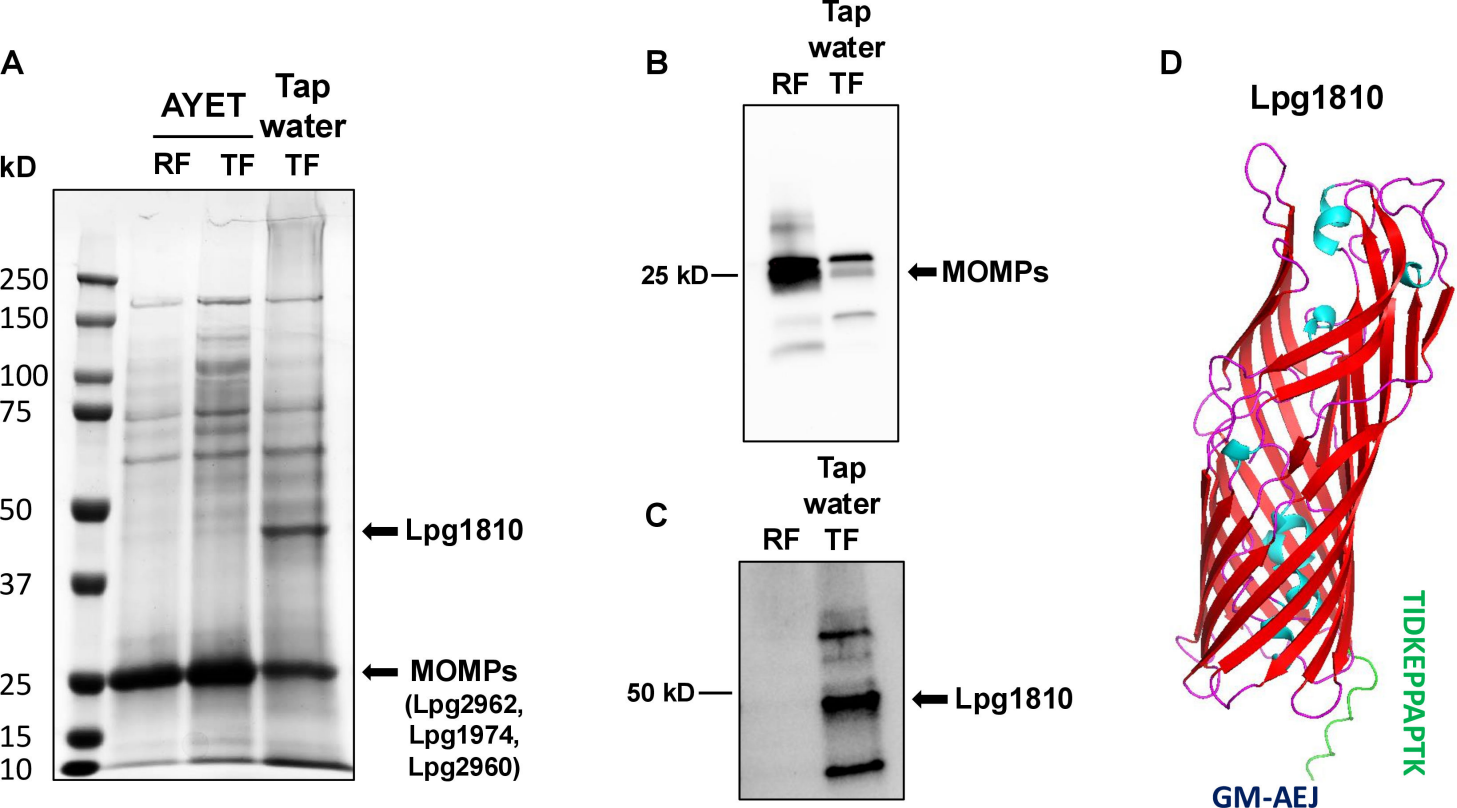
β-barrel tethering is not limited to the survival variants and an OM long-chain fatty acid transporter is tethered to PG in *L. pneumophila*. (**A**) SDS-PAGE showing PG-attached proteins in the replicating (RF) and survival (TF) variants of *L. pneumophila* harvested from AYET broth or tap water. AYET TF refers to *L. pneumophila* culture grown to post-exponential transmissive phase in AYET broth. PG was extracted from the respective variants and run in SDS-PAGE followed by identification of SYPRO Ruby-stained protein bands by standard mass spectrometry analysis. (**B**) Immunoblot images detecting MOMPs attached to PG in RFs and TFs of *L. pneumophila*. PG was extracted from RFs and TFs (tap water) and probed with anti-MOMP antibodies. (**C**) Immunoblot images detecting Lpg1810 attached to PG in TFs of *L. pneumophila*. PG was extracted from RFs and TFs (tap water) and probed with anti-Lpg1810 antibody. (**D**) AlphaFold prediction showing C-terminal periplasmic tail (KTPAPPEKDIT, green colored) of OM long-chain fatty acid transporter (Lpg1810) of *L. pneumophila*. Byos predicts C-terminal periplasmic tail (KTPAPPEKDIT) of Lpg1810 (UniProt: Q5ZUI8) gets tethered to PG (GM-AEJ) through lysine (**K**) linkage (GM-AEJKTPAPPEKDIT).

This study corroborates a recent study (16) that β-barrel tethering exists only in the survival variants but not in the replicating variants of *C. burnetii* (Fig. 1D; Table S2). Contrary to our expectation, in *L. pneumophila*, we found tethering of β-barrel OM proteins, MOMPs, in both the replicating and survival variants (Fig. 1F; Fig. S2 and S3; Table S3), suggesting that β-barrel tethering is not limited to the survival variants. This was further validated by SDS-PAGE and immunoblot analyses of PG-attached proteins, where MOMPs (Lpg2961, Lpg1974, and Lpg2960) were identified and/or detected in both the replicating and survival variants of *L. pneumophila* (Fig. 6A and B). Furthermore, compared to the recent report (16), we identified additional β-barrel OM proteins, Cbu1814 (GM-AEJGGIEVPH) and Lpg2942 (GM-AEJGTVGEAQQDGK), tethered to PG in *C. burnetii* and *L. pneumophila*, respectively (Tables S2 and S3), which requires the further validation.

Interestingly, we found an OM long-chain fatty acid transporter, Lpg1810 (UniProt: Q5ZUI8), belonging to the OmpP1/FadL family, tethered to PG (GM-AEJKTPAPPEKDIT) in *L. pneumophila* (Fig. 6A, C and D; Table S3). Furthermore, tethering of Lpg1810 was restricted to the survival variants harvested from tap water (Fig. 6A and C), suggesting a potential role of this tethering in bacterial survival under nutrient-limited environments. AlphaFold predicts a C-terminal periplasmic tail (KTPAPPEKDIT) of Lpg1810 (Fig. 6D) and Byos analysis of PG-tethered proteins determines a lysine (K) residue of Lpg1810 covalently attaching *m*DAP (J) residue of PG (GM-AEJKTPAPPEKDIT) (Fig. S9). Taking advantage of UniProt accessible eggNOG phylogenomic database (http://eggnog6.embl.de/search/ogs/COG2067/), we searched for the orthologs of Lpg1810, finding them to be widespread in bacteria, particularly in proteobacteria, including alpha-, beta-, and gamma-proteobacteria. The alignment of orthologs revealed that the fatty acid transporters with *Legionella*-like C-terminal periplasmic tail are common in Hyphomicrobiales (data not shown), particularly in the Methylobacteriaceae family of bacteria that are known as opportunistic pathogens to humans and survive in the water systems like *Legionella* (51). For instance, AlphaFold predicted residues “WDAPAAVAPAPLVRKF” and “DPVPALEPYK” as C-terminal periplasmic tails of OM long-chain fatty acid transporters in *Methylorubrum extorquens* (UniProt: C5B300, Fig. S10A) and *Rhodomicrobium vannielii* (UniProt: E3I2K7, Fig. S10B), respectively. This novel finding revealing that fatty acid transporter is tethered to PG in bacteria requires further investigation to understand its structural and functional significance in bacterial survival physiology. Understanding these kinds of unique transporters could help to reveal a potentially shared mechanism of bacterial survival in nutrient or growth-limited environments.

## DISCUSSION

**Fig 7:**
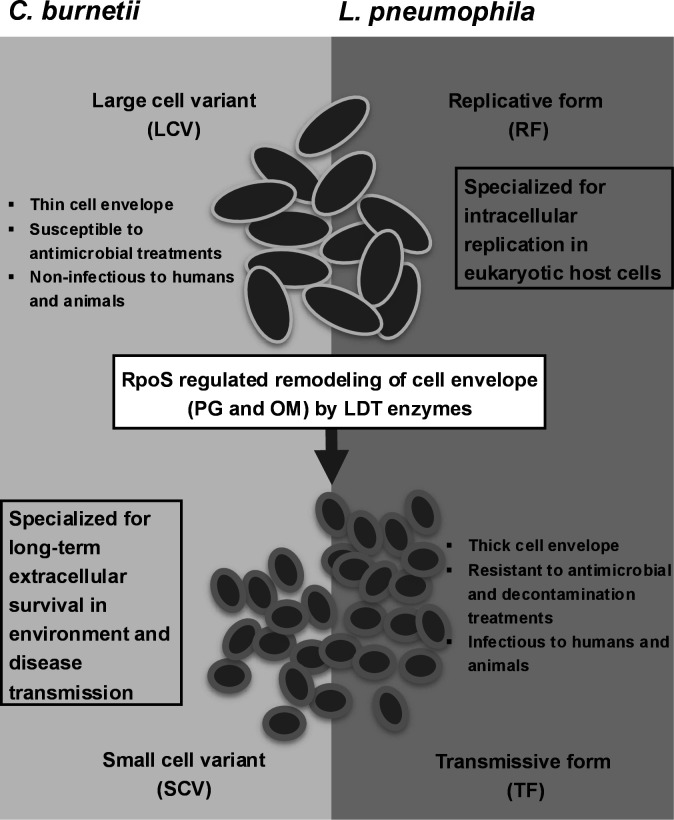
A model figure depicting developmental transitions in *C. burnetii* and *L. pneumophila*. Replicating variants (LCV: large-cell variant and RF: replicative form) generally have thin cell envelopes, and are non-infectious, susceptible to antimicrobial treatments, and specialized for intracellular replication in host cells. Survival variants (SCV: small-cell variant and TF: transmissive form) generally have thick cell envelope and are infectious, resistant to antimicrobial and decontamination treatments, and specialized for long-term survival in the environment and host. The cell envelope PG and outer membrane (OM) remodeling mediated by LDT enzymes and significantly regulated by RpoS, a stationary-phase/stress sigma factor, facilitates the developmental transitions in *C. burnetii* and *L. pneumophila*.

Here, we characterized the cell envelope architecture of replicating and survival variants of *C. burnetii* and *L. pneumophila*. We uncovered that LDTs are upregulated in the survival variants with enrichment of LDT-catalyzed PG structures*,* demonstrating the role of LDTs in the developmental transitions and survival of *C. burnetii* and *L. pneumophila* (Fig. 7). We observed the activation of multiple LDTs in the survival variants, suggesting that LDTs could have redundant and/or specific functions. Other important bacterial pathogens such as *E. coli* (6–9), *Pseudomonas aeruginosa* (52), and *Mycobacterium tuberculosis* (53, 54) also encode multiple LDTs (3–6) with both redundant and specific functions. Consistent with our study, four LDTs (*lpg*1386, *lpg*1336, *lpg*2641, and *lpg*0910) were found upregulated in *L. pneumophila* incubated in the artificial freshwater medium Fraquil (55). However, another study found *lpg*1697 upregulated when *L. pneumophila* is subjected to long-term incubation in tap water at 42°C (56). We did not detect the activation of *lpg*1697, which could be due to a difference in incubation temperature, suggesting that bacteria can upregulate a specific LDT when exposed to thermal stress. To support this, LdtD in *E. coli* is particularly activated during a cold shock to remodel the cell envelope (57). In line with LDTs activation, carbapenem antibiotics were found effective against the survival variants*,* which warrants testing the efficacy of carbapenems to treat chronic infections caused by these pathogens, particularly chronic Q fever (21, 58), as well as LDT-targeting chemical agents such as copper (59) to decontaminate the environmental reservoirs during outbreaks. Importantly, recent studies have reported an unusual role of LDTs (35, 36) and the discovery of new families of LDTs (60, 61), highlighting the need for further exploration of LDTs and their roles in non-model bacteria.

The LDTs *cbu*0318 and *lpg*1386 were most upregulated in the survival variants of *C. burnetii* and *L. pneumophila*, respectively. A recent study showed that *cbu*0318 is involved in β-barrel tethering in *C. burnetii* (16), and the current study revealed that *lpg*1386 is involved in 3-3 cross-linking in *L. pneumophila* and mediates resistance to β-lactam antibiotics. Our finding of 3-3 cross-linker-mediating β-lactam resistance is consistent with other studies in *E. coli* (11, 12) and *M. tuberculosis* (53). The *lpg*1386 knockout is not susceptible to carbapenems, suggesting this LDT is one of the targets of carbapenems in *L. pneumophila*. Similar to our finding, carbapenems effectively inhibit LDTs in other bacterial pathogens, including *M. tuberculosis*, *M. abscessus*, *Clostridium difficile*, and *Enterococcus faecium* (62–65). It is intriguing to understand the roles of other LDTs upregulated in the survival variants of both of these pathogens and how these LDTs function in a concerted way. We provided evidence that LDT-catalyzed PG remodeling is significantly regulated by an RpoS sigma factor. This is consistent with other published studies (27, 48). RpoS regulates the genes associated with SCV development in *C. burnetii* that includes genes involved in stress responses, amino acid transport, cell wall remodeling, and type IVB effector secretion system (27). In *L. pneumophila*, RpoS regulates the expression of transmission traits along with other regulators, including the LetA/LetS two-component system (48, 49), which might explain the partial regulation of PG remodeling by RpoS in this bacterium.

We showed that the host intracellular growth environment significantly elevates the LDT-catalyzed PG remodeling and alters the tethering systems in *C. burnetii*. The activation of additional LDTs is suggestive of different stressors encountered by the bacteria in the host cells as compared to the axenic environment. The increased β-barrel tethering and 3-3 cross-linking induced by the host environment in *C. burnetii* could make this bacterium hyperstable and resistant, as β-barrel tethering is considered important for the cell envelope stabilization in bacteria (17, 18) and LDT cross-links contribute to the stress resistance (5). Importantly, LimB tethering was absent in the survival variants derived from the host cells. LimB is reported to be involved in the binding and acquisition of metals in *C. burnetii* (66) and iron is required for the intracellular replication in the host cells (67). Therefore, it is possible that LimB tethering is essential for metal acquisition when bacteria are replicating, that is, the LCV stage, but not required during the non-replicating SCV stage. We observed increased deacetylated PG sugars in the survival variants that are consistent with other studies (23, 68). The PG deacetylation protects the bacteria from host immune components and also aids in immune evasion, thereby promoting bacterial survival (4). On the other hand, fewer anhydro PG sugars were observed in the survival variants, which is suggestive of longer glycan chains and more rigid PG structure (69) leading to stronger bacterial resistance against environmental stresses. This is further supported by a recent study (70) where an inverse correlation between LDT activation and the level of anhydro PG sugars was reported.

Contrary to expectation (16), our data suggest that β-barrel tethering is not specific to the survival variants in *L. pneumophila*. The MOMPs tethering in *L. pneumophila* might activate them as porins (71, 72) in addition to providing the stability to cell envelope. We discovered a unique OM long-chain fatty acid transporter (Lpg1810) tethered to PG in the survival variants of *L. pneumophila*. It is reported that Lpg1810 is involved in fatty acid uptake (73) and subsequent biosynthesis of poly-3-hydroxybutyrate, an intracellular carbon storage molecule, to facilitate bacterial survival under nutrient-limited conditions (74). Furthermore, a recent study uncovered that *L. pneumophila* exploits host cell fatty acids to promote expansion of the replication vacuole and bacterial growth, suggesting *L. pneumophila*’s potential to utilize the fatty acids as a nutrient substrate (75). These data together suggest that the physiological role of PG-OM tethering might not be only to provide the cell envelope stability but also to aid in the nutrient uptake in bacteria. To support this, a recent study reported that OmpM in *Veillonella parvula* tethers PG and also functions as a nutrient-uptake channel (76). Furthermore, another study pointed out the role of tethering in nutrient homeostasis in plant cells (77). The Lpg1810 homologs are common in the Methylobacteriaceae family of bacteria that are enriched in LDTs (e.g., ~28 LDTs in *M. extorquens*, data not published) and also survive in the water systems (51), as does *Legionella*, thus suggesting a potentially conserved mechanism of bacterial survival in the nutrient-limited environments. Unlike in *C. burnetii* and the currently available knowledge that NCAAs incorporation in PG is a stationary-phase phenomenon (10), the higher incorporation of NCAAs was observed in the exponential replicating variants of *L. pneumophila*, which requires further investigation.

In conclusion, our study determined that LDT-mediated cell envelope remodeling enables developmental transitions and survival in *C. burnetii* and *L. pneumophila*. Our future studies will be focused on delineating the additional roles of LDTs and understanding the functional relevance of β-barrel, LimB, and Lpg1810 tetherings in *C. burnetii* and *L. pneumophila*.

## Data Availability

The authors confirm that the data supporting the findings of this study are available within the article and supplementary materials.

## SUPPLEMENTAL MATERIAL

Supplemental figures jb.00247-24-s0001.pdf Fig. S1 to S10.

Table S1 jb.00247-24-s0002.xlsx List of primers used in this study.

Table S2 jb.00247-24-s0003.xlsx Relative abundance (%) of PG structures in SCV and LCV variants of *C. burnetii*.*J: *meso*DAP.

Table S3 jb.00247-24-s0004.xlsx Relative abundance (%) of PG structures in RF and TF variants of *L. pneumophila*.*J: *meso*DAP.

Table S4 jb.00247-24-s0005.xlsx Fold change in expression.

Table S5 jb.00247-24-s0006.xlsx MICs comparison between RFs and TFs of *L. pneumophila*.

Table S6 jb.00247-24-s0007.xlsx Relative abundance (%) of PG structures in SCV variants of WT and rpoS knock-out strains of *C. burnetii.**J: *meso*DAP.

Table S7 jb.00247-24-s0008.xlsx Relative abundance (%) of PG structures in TF variants of WT and rpoS knock-out strains of *L. pneumophila.**J: *meso*DAP.

Table S8 jb.00247-24-s0009.xlsx Relative abundance (%) of PG structures in SCV variants of *C. burnetii* harvested from infected oTr1 cells and ACCM-D broth.*J: *meso*DAP.

ASM does not own the copyrights to Supplemental Material that may be linked to, or accessed through, an article. The authors have granted ASM a non-exclusive, world-wide license to publish the Supplemental Material files. Please contact the corresponding author directly for reuse.
