# Supplementary material for: LD-transpeptidase-mediated cell envelope remodeling enables developmental transitions and survival in Coxiella burnetii and Legionella pneumophila
Source: J Bacteriol. 2025 Jan 23;207(2):e00247-24. doi: 10.1128/jb.00247-24 (PMC11841132; doi:10.1128/jb.00247-24)

Supplementary figure legends

Fig. S1. Structural annotation of the PG peaks of replicating (LCV) and survival (SCV) variants of *C*. *burnetii* as determined by LC-MS/MS analysis. Peak structures in bold indicate the peaks unique to the SCV variants (G: GlcNAc, M: MurNAc, J: *m*DAP, DeAc: deacetylated, Anh: anhydro, Double_Anh: double anhydro, others are single-letter code for amino acids).

Fig. S2. Structural annotation of the PG peaks of replicating (RF) and survival (TF) variants of *L*. *pneumophila* as determined by LC-MS/MS analysis. Peak structures in bold indicate the peaks unique to the TF variants (G: GlcNAc, M: MurNAc, J: *m*DAP, DeAc: deacetylated, Anh: anhydro, others are single-letter code for amino acids).

Fig. S3. Magnified view of the relative abundance of β-barrel and Lpg1810 tethering in the replicating (RF) and survival (TF) variants of *L*. *pneumophila*. ***$P<0.01$, ****$P<0.001$, multiple unpaired t-tests.

Fig. S4. Structural annotation of the PG peaks of survival (SCV) variants of wild-type (WT) and *rpoS* knock-out (Δ*rpoS*) strains of *C*. *burnetii* as determined by LC-MS/MS analysis. Peak structures in bold indicate the peaks present only in WT strain but not in Δ*rpoS* strain (G: GlcNAc, M: MurNAc, J: *m*DAP, DeAc: deacetylated, Anh: anhydro, Double_Anh: double anhydro, others are single-letter code for amino acids).

Fig. S5. Structural annotation of the PG peaks of survival (TF) variants of wild-type (WT) and *rpoS* knock-out (Δ*rpoS*) strains of *L*. *pneumophila* as determined by LC-MS/MS analysis. Peak structures in bold indicate the peaks present only in WT strain but not in Δ*rpoS* strain (G: GlcNAc, M: MurNAc, J: *m*DAP, DeAc: deacetylated, DeAc_Anh: deacetylated and anhydro, Double_Anh: double anhydro, others are single-letter code for amino acids).

Fig. S6. Structural annotation of the PG peaks of survival (SCV) variants of *C. burnetii* harvested from ACCM-D broth and oTr1 cells as determined by LC-MS/MS analysis. Peak structures in bold indicate the peaks whose abundance is drastically changed in SCVs from oTr1 cells compared to ACCM-D broth (G: GlcNAc, M: MurNAc, J: *m*DAP, DeAc: deacetylated, Anh: anhydro, Double_Anh: double anhydro, others are single-letter code for amino acids).

Fig. S7. Structural annotation of the PG peaks of survival (TF) variants of wild-type (WT) and *lpg*1386 knock-out (Δ*lpg*1386) strains of *L. pneumophila* as determined by LC-MS/MS analysis. Peak structures in bold indicate the peaks absent in Δ*lpg*1386 strain (G: GlcNAc, M: MurNAc, J: *m*DAP, DeAc: deacetylated, Anh: anhydro, others are single-letter code for amino acids).

Fig. S8. Viable bacterial counts compared between wild-type and Δ*lpg*1386 strains at 6 days post-incubation in tap water determined by ten-fold serial dilution in CYET agar plate.

Fig. S9. Byos identification of traces of C-terminal periplasmic tail (KTPAPPEKDIT) of long-chain fatty acid transporter (Lpg1810) attached to PG (GM-AEJ) through lysine (K) linkage (GM-AEJKTPAPPEKDIT). For the upper panel, PG modification: GM (GlcNAc-MurNAc) was allowed, which identified GM covalently attached to tripeptide (AEJ) and C-terminal periplasmic trace (KTPAPPE) of Lpg1810 through lysine (K) linkage (GM-AEJKTPAPPE). For the lower panel, PG modification: GM-AEJ was allowed, which identified GM-AEJ covalently attached to C-terminal periplasmic trace (KTPAPPEKDIT) of Lpg1810 through threonine (T) linkage (GM-AEJKTPAPPEKDIT).

Fig. S10. (A&B) AlphaFold predictions showing C-terminal periplasmic tails (green colored) of long-chain fatty acid OM transporters in *Methylorubrum extorquens* (UniProt: C5B300) and *Rhodomicrobium vannielii* (UniProt: E3I2K7), respectively.

Fig. S1

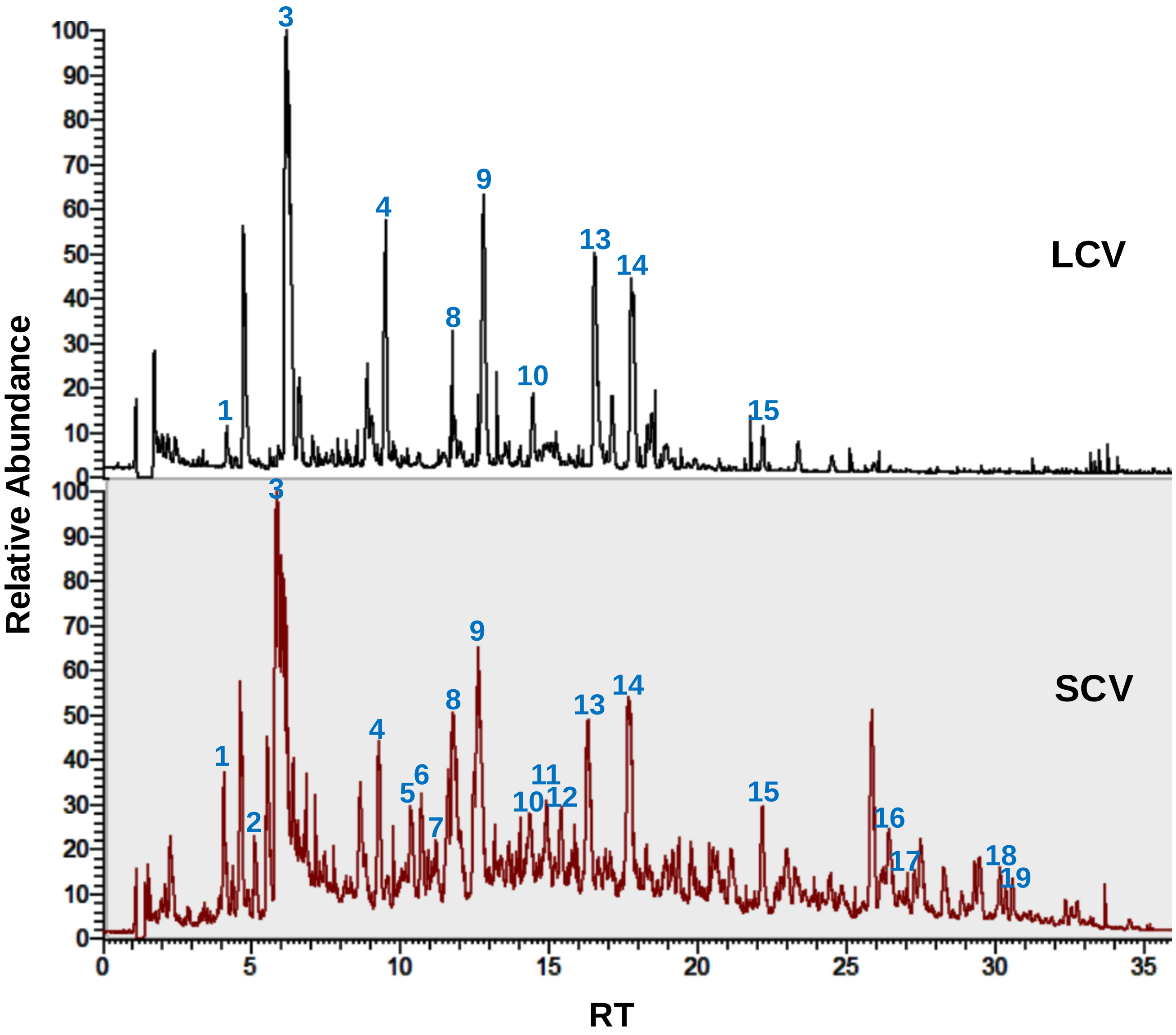

#peak: identified structure
1: GM-AEJ
2: GM-AEJA (DeAc)
3: GM-AEJA
4: GM-GM-AEJA
5: GM-AEJG-GM-AEJ
6: GM-AEJ-GM-AEJ
7: GM-AEJG-GM-AEJA
8: GM-AEJ-GM-AEJA
9: GM-AEJA-GM-AEJA
10: GM-GM-AEJA-GM-AEJA
11: GM-AEJ-GM-AEJ-GM-AEJA
12: GM-AEJA-GM-AEJ-GM-AEJA
13: GM-AEJAKL
14: GM-AEJA-GM-AEJA (Anh)
15: GM-AEJA-GM-AEJA (Double_Anh)
16: GM-AEJGGPDYVPAPS
17: GM-AEJAGGPDYVPAPS
18: GM-AEJGGPIDM
19: GM-AEJGGPDYVPAPSY

Fig. S2

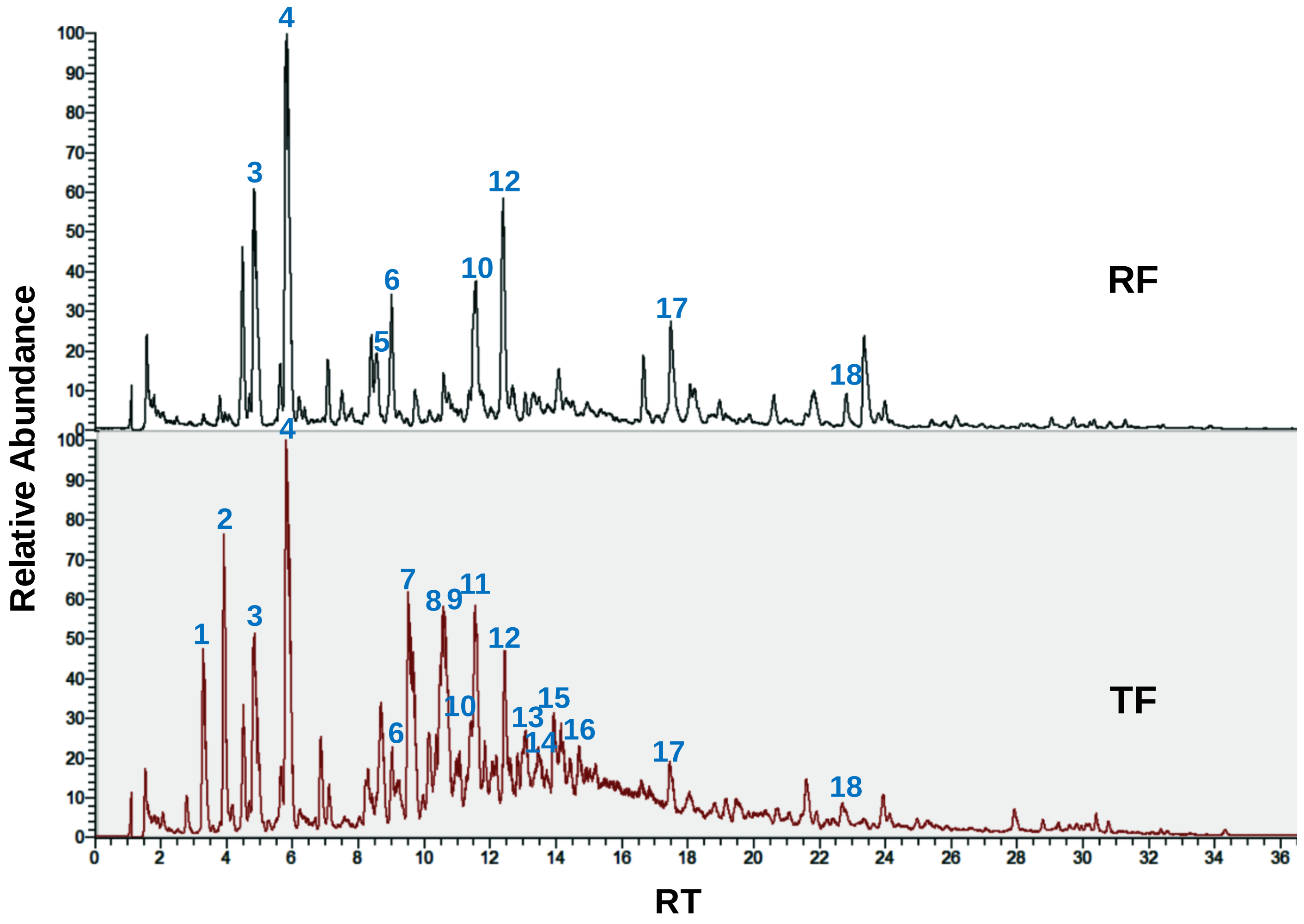

#peak: **identified structure**

1: GM-AEJ (DeAc)

2: GM-AEJ

3: GM-AEJA (DeAc)

4: GM-AEJA

5: GM-AEJGTVGEA

6: GM-GM-AEJA

7: GM-AEJ-GM-AEJ (DeAc)

8: GM-AEJ-GM-AEJ

9: GM-AEJ-GM-AEJA (DeAc)

10: GM-AEJA-GM-AEJA (DeAc)

11: GM-AEJ-GM-AEJA

12: GM-AEJA-GM-AEJA

13: GM-AEJ-GM-AEJ-GM-AEJ (DeAc)

14: GM-AEJ-GM-AEJ-GM-AEJ

15: GM-AEJ-GM-AEJ-GM-AEJA (DeAc)

16: GM-AEJ-GM-AEJ-GM-AEJA

17: GM-AEJA-GM-AEJA (Anh)

18: GM-AEJGTMGPV

Fig. S3

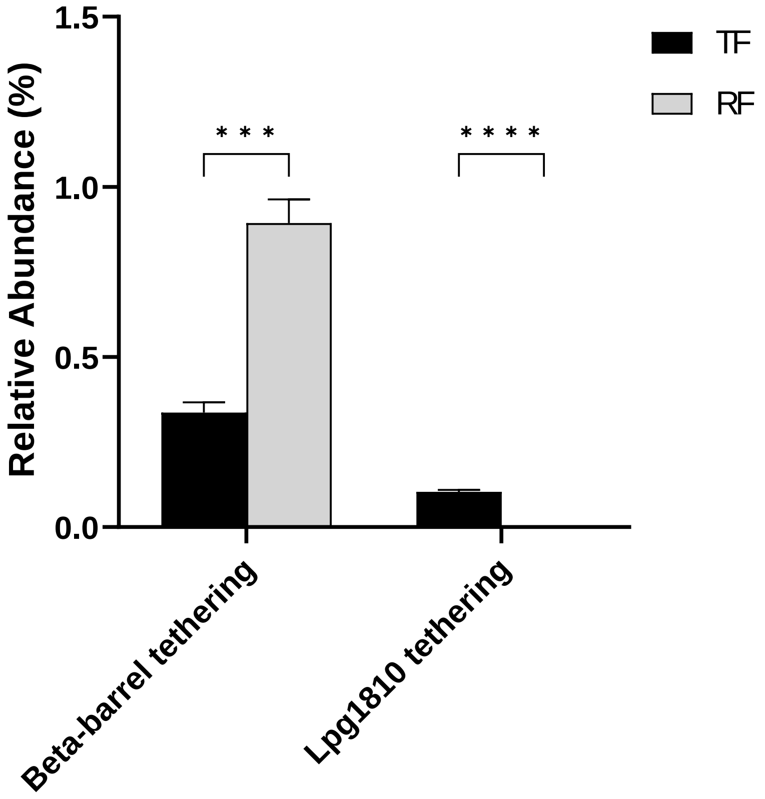

1.5
1.0
0.5
0.0
Relative Abundance (%)
* * *
* * * *
TF
RF
Beta-barrel tethering
Lpg1810 tethering

Fig. S4

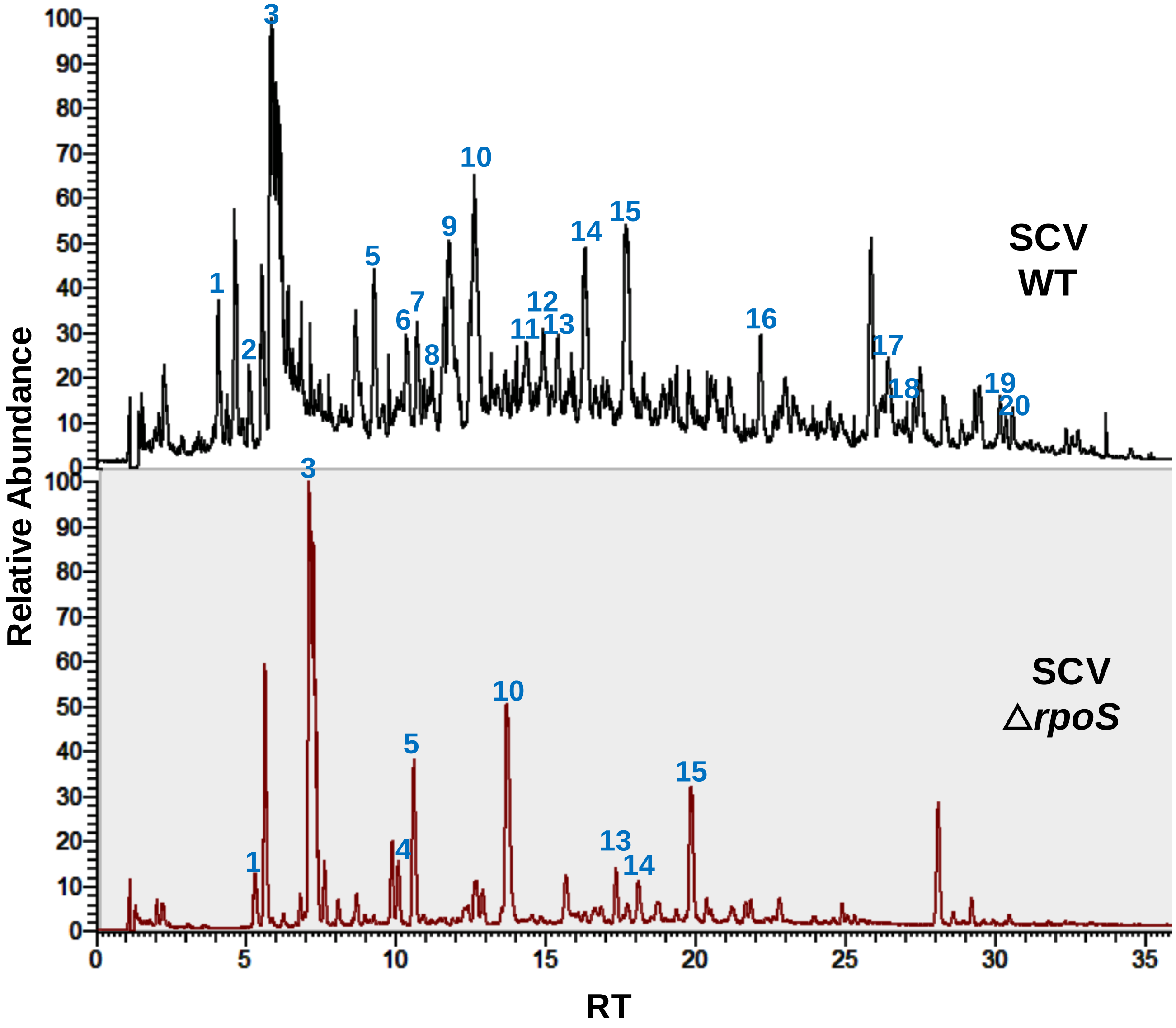

#peak: identified structure

1: GM-AEJ
2: GM-AEJA (DeAc)
3: GM-AEJA
4: GM-AEJA-GM-AEJA (Amidase product)
5: GM-GM-AEJA
6: GM-AEJG-GM-AEJ
7: GM-AEJ-GM-AEJ
8: GM-AEJG-GM-AEJA
9: GM-AEJ-GM-AEJA
10: GM-AEJA-GM-AEJA
11: GM-GM-AEJA-GM-AEJA
12: GM-AEJ-GM-AEJ-GM-AEJA
13: GM-AEJA-GM-AEJ-GM-AEJA
14: GM-AEJAKL
15: GM-AEJA-GM-AEJA (Anh)
16: GM-AEJA-GM-AEJA (Double_Anh)
17: GM-AEJGGPDYVPAPS
18: GM-AEJAGGPDYVPAPS
19: GM-AEJGGPIDM
20: GM-AEJGGPDYVPAPSY

Fig. S5

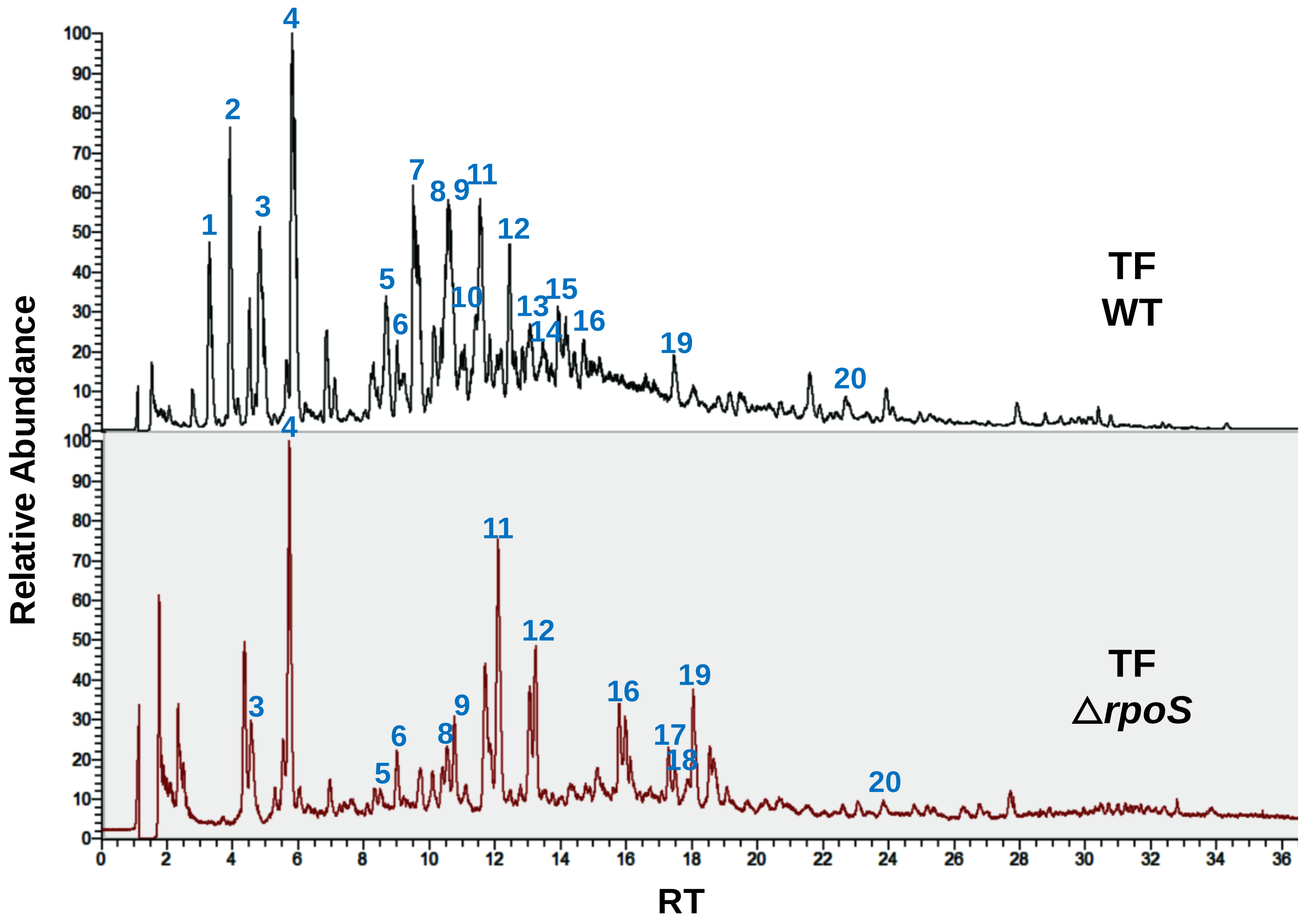

#peak: identified structure

1: GM-AEJ (DeAc)
2: GM-AEJ
3: GM-AEJA (DeAc)
4: GM-AEJA
5: GM-AEJA-GM-AEJA (Amidase Product)
6: GM-GM-AEJA
7: GM-AEJ-GM-AEJ (DeAc)
8: GM-AEJ-GM-AEJ
9: GM-AEJ-GM-AEJA (DeAc)
10: GM-AEJA-GM-AEJA (DeAc)
11: GM-AEJ-GM-AEJA
12: GM-AEJA-GM-AEJA
13: GM-AEJ-GM-AEJ-GM-AEJ (DeAc)
14: GM-AEJ-GM-AEJ-GM-AEJ
15: GM-AEJ-GM-AEJ-GM-AEJA (DeAc)
16: GM-AEJ-GM-AEJ-GM-AEJA
17: GM-AEJA-GM-AEJA (DeAc_Anh)
18: GM-AEJ-GM-AEJA (Anh)
19: GM-AEJA-GM-AEJA (Anh)
20: GM-AEJGTMGPV

Fig. S6

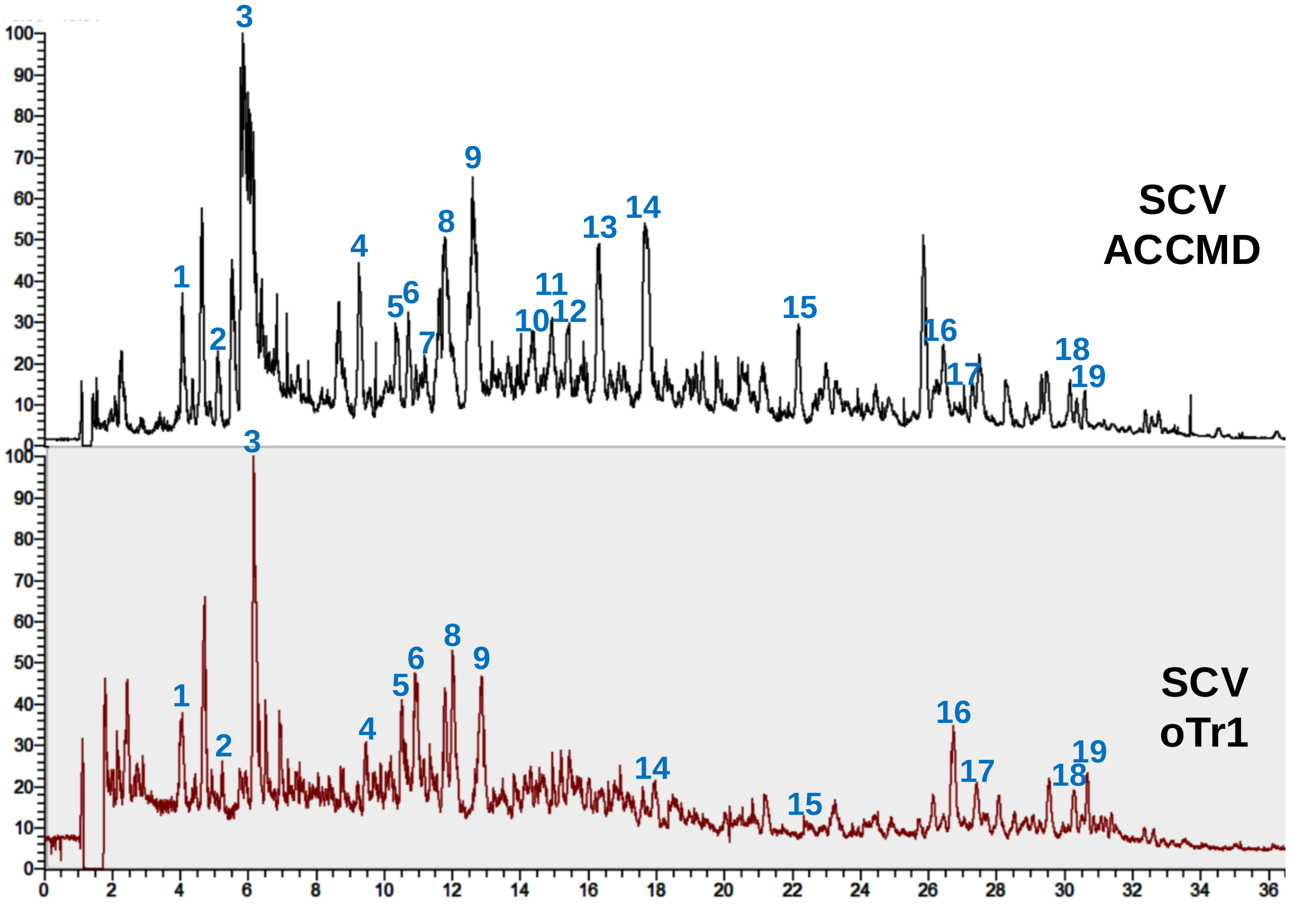

#peak: identified structure

1: GM-AEJ
2: GM-AEJA (DeAc)
3: GM-AEJA
4: GM-GM-AEJA
5: GM-AEJG-GM-AEJ
6: GM-AEJ-GM-AEJ
7: GM-AEJG-GM-AEJA
8: GM-AEJ-GM-AEJA
9: GM-AEJA-GM-AEJA
10: GM-GM-AEJA-GM-AEJA
11: GM-AEJ-GM-AEJ-GM-AEJA
12: GM-AEJA-GM-AEJ-GM-AEJA
13: GM-AEJAKL
14: GM-AEJA-GM-AEJA (Anh)
15: GM-AEJA-GM-AEJA (Double_Anh)
16: GM-AEJGGPDYVPAPS
17: GM-AEJAGGPDYVPAPS
18: GM-AEJGGPIDM
19: GM-AEJGGPDYVPAPSY

Fig. S7

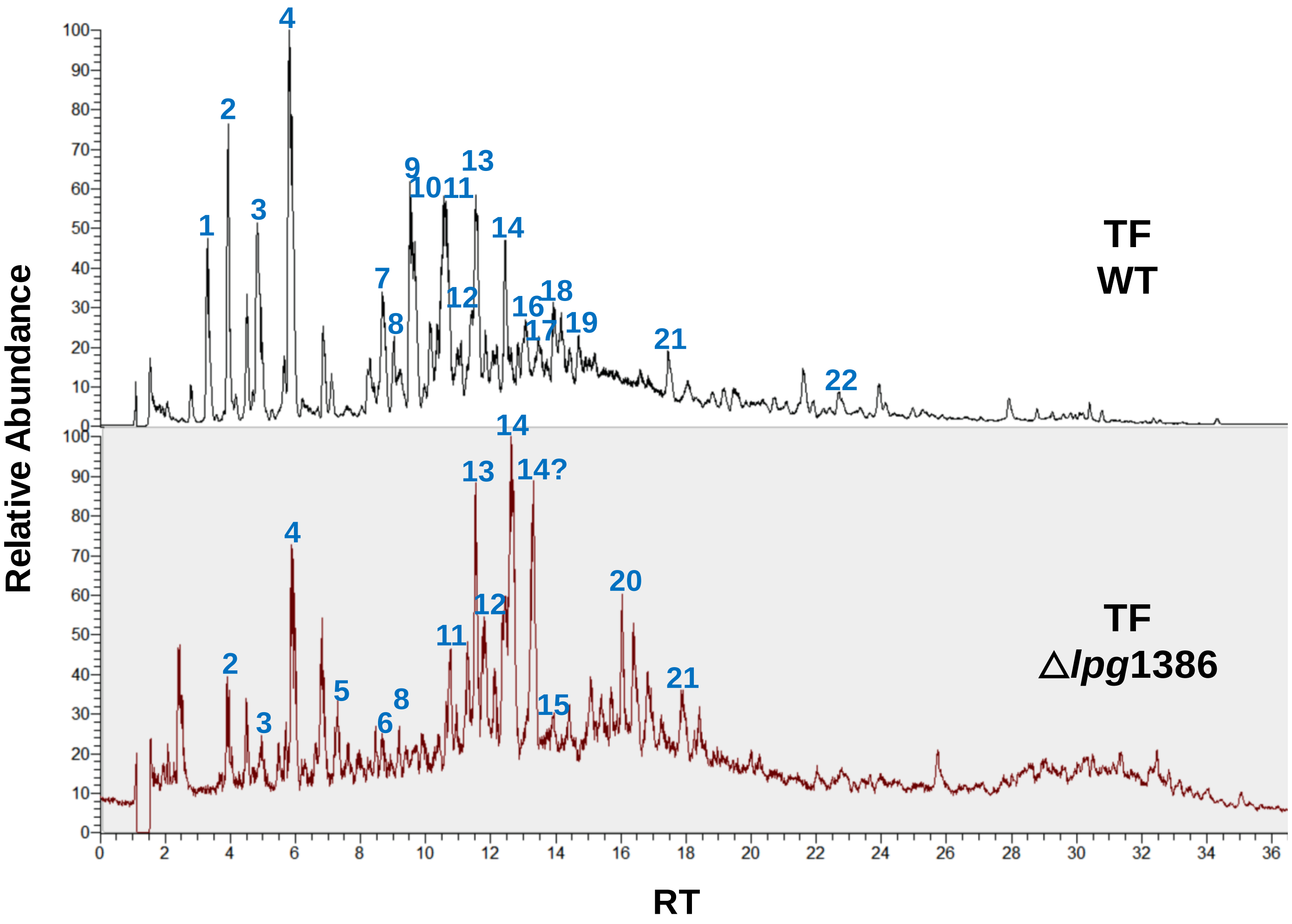

#peak: identified structure

1: GM-AEJ (DeAc)
2: GM-AEJ
3: GM-AEJA (DeAc)
4: GM-AEJA
5: GM-AEJGTVGE
6: GM-AEJGTVGEA
7: GM-AEJA-GM-AEJA (Amidase Product)
8: GM-GM-AEJA
9: GM-AEJ-GM-AEJ (DeAc)
10: GM-AEJ-GM-AEJ
11: GM-AEJ-GM-AEJA (DeAc)
12: GM-AEJA-GM-AEJA (DeAc)
13: GM-AEJ-GM-AEJA
14: GM-AEJA-GM-AEJA
15: GM-AEJAA-GM-AEJA
16: GM-AEJ-GM-AEJ-GM-AEJ (DeAc)
17: GM-AEJ-GM-AEJ-GM-AEJ
18: GM-AEJ-GM-AEJ-GM-AEJA (DeAc)
19: GM-AEJ-GM-AEJ-GM-AEJA
20: GM-AEJA-GM-AEJA-GM-AEJA
21: GM-AEJA-GM-AEJA (Anh)
22: GM-AEJGTMGPV

Fig. S8

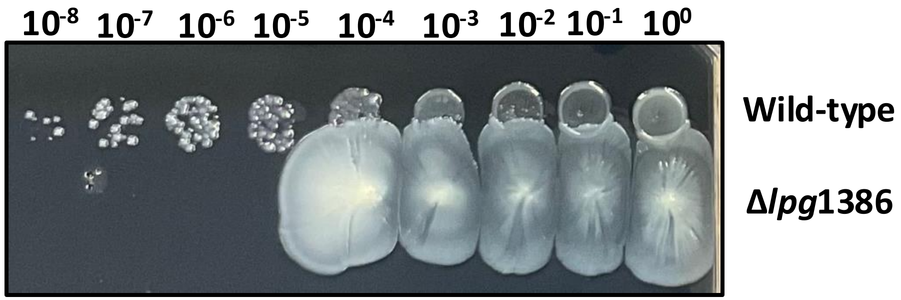

Fig. S9

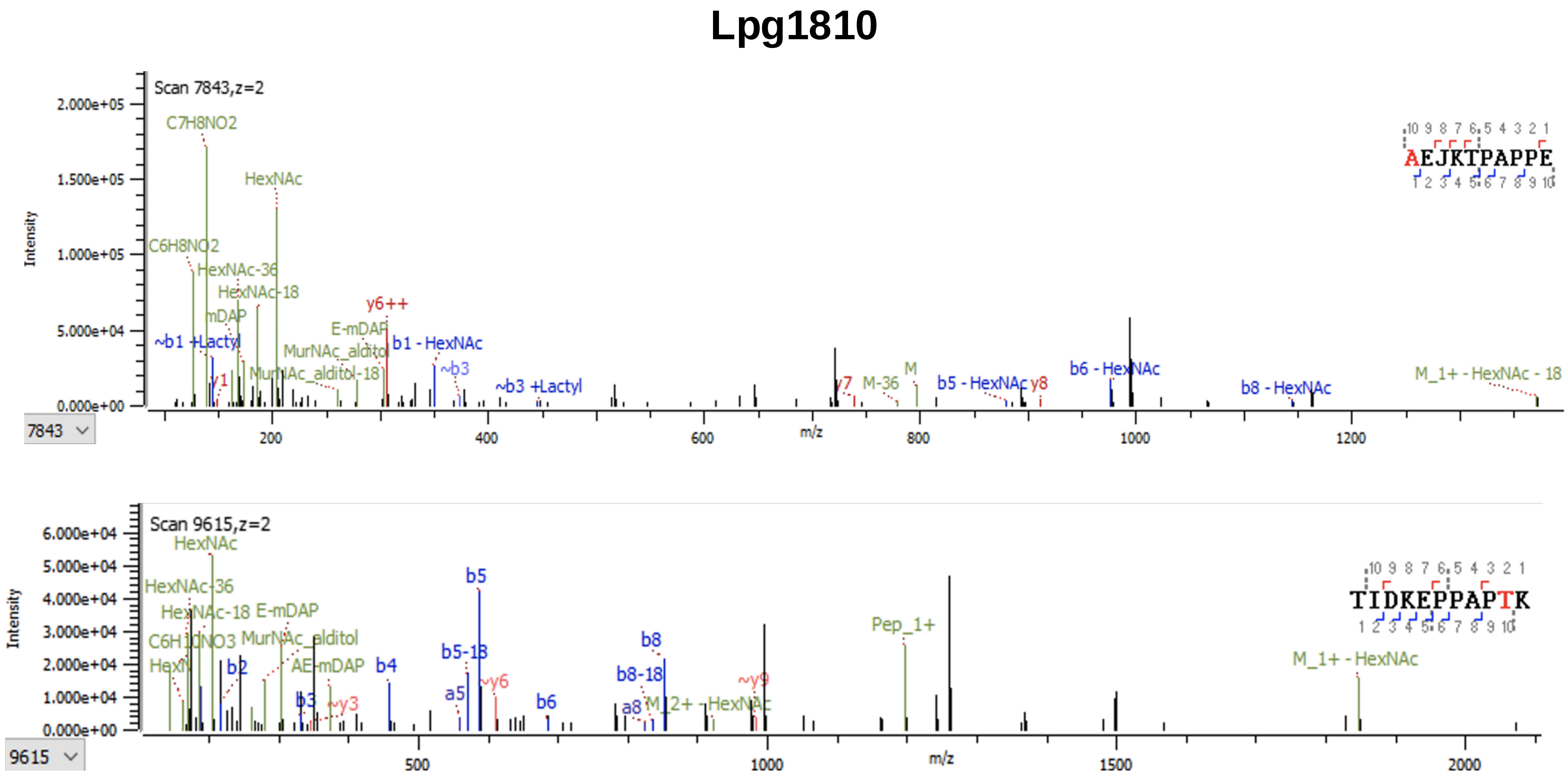
Lpg1810
Scan 7843,z=2
AEJKTPAPPE
Intensity
m/z
7843
Scan 9615,z=2
TIDKEPPAPTK
Intensity
m/z
9615

Fig. S10

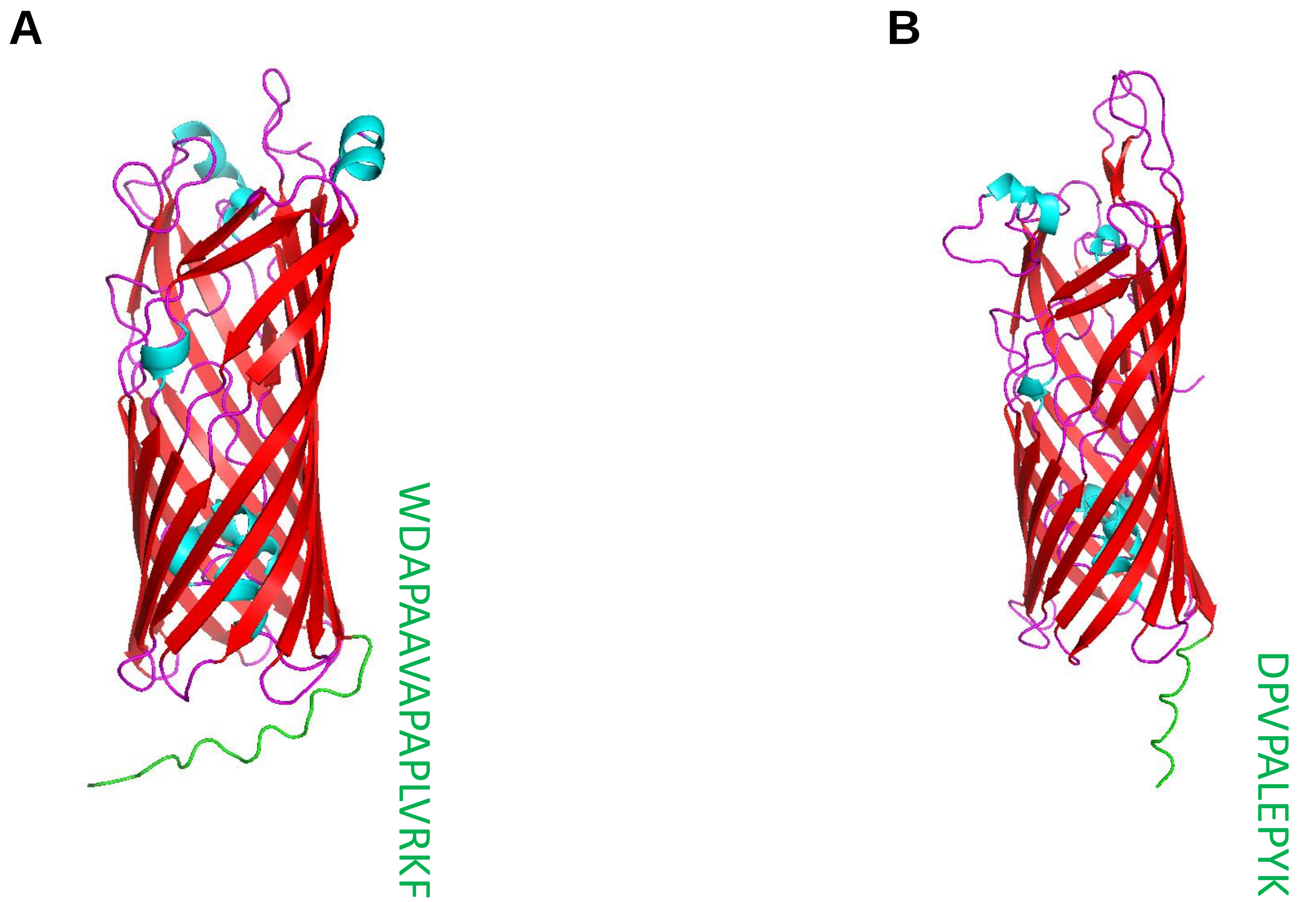

Supplement: Supplemental figures — Fig. S1 to S10. [file jb.00247-24-s0001.pdf]
